# Diatom Proteomics Reveals Unique Acclimation Strategies to Mitigate Fe Limitation

**DOI:** 10.1371/journal.pone.0075653

**Published:** 2013-10-16

**Authors:** Brook L. Nunn, Jessica F. Faux, Anna A. Hippmann, Maria T. Maldonado, H. Rodger Harvey, David R. Goodlett, Philip W. Boyd, Robert F. Strzepek

**Affiliations:** 1 Department of Genome Sciences, University of Washington, Seattle, Washington, United States of America; 2 Medicinal Chemistry Department, University of Washington, Seattle, Washington, United States of America; 3 University of Maryland Center for Environmental Science, Chesapeake Biological Laboratory, Solomons, Maryland, United States of America; 4 Department of Earth, Ocean and Atmospheric Sciences, University of British Columbia, Vancouver, British Columbia, Canada; 5 Department of Ocean, Earth and Atmospheric Science, Old Dominion University, Norfolk, Virginia, United States of America; 6 NIWA Centre for Chemical and Physical Oceanography, Department of Chemistry, University of Otago, Dunedin, New Zealand; 7 Research School of Earth Sciences, The Australian National University, Canberra, Australia; Laurentian University, Canada

## Abstract

Phytoplankton growth rates are limited by the supply of iron (Fe) in approximately one third of the open ocean, with major implications for carbon dioxide sequestration and carbon (C) biogeochemistry. To date, understanding how alteration of Fe supply changes phytoplankton physiology has focused on traditional metrics such as growth rate, elemental composition, and biophysical measurements such as photosynthetic competence (F_v_/F_m_). Researchers have subsequently employed transcriptomics to probe relationships between changes in Fe supply and phytoplankton physiology. Recently, studies have investigated longer-term (i.e. following acclimation) responses of phytoplankton to various Fe conditions. In the present study, the coastal diatom, *Thalassiosira pseudonana*, was acclimated (10 generations) to either low or high Fe conditions, i.e. Fe-limiting and Fe-replete. Quantitative proteomics and a newly developed proteomic profiling technique that identifies low abundance proteins were employed to examine the full complement of expressed proteins and consequently the metabolic pathways utilized by the diatom under the two Fe conditions. A total of 1850 proteins were confidently identified, nearly tripling previous identifications made from differential expression in diatoms. Given sufficient time to acclimate to Fe limitation, *T. pseudonana* up-regulates proteins involved in pathways associated with intracellular protein recycling, thereby decreasing dependence on extracellular nitrogen (N), C and Fe. The relative increase in the abundance of photorespiration and pentose phosphate pathway proteins reveal novel metabolic shifts, which create substrates that could support other well-established physiological responses, such as heavily silicified frustules observed for Fe-limited diatoms. Here, we discovered that proteins and hence pathways observed to be down-regulated in short-term Fe starvation studies are constitutively expressed when *T. pseudonana* is acclimated (i.e., nitrate and nitrite transporters, Photosystem II and Photosystem I complexes). Acclimation of the diatom to the desired Fe conditions and the comprehensive proteomic approach provides a more robust interpretation of this dynamic proteome than previous studies.

## Introduction

Many field studies have demonstrated that phytoplankton stocks across the world's oceans are frequently limited by Fe supply [1]–[4]. The adverse effects of low Fe concentrations on primary production are well established in ∼30% of the world's oceans, the so-called High Nitrate Low Chlorophyll (HNLC) regions [5]–[7]. The widespread Fe limitation of phytoplankton in HNLC waters has major implications for the ocean C cycle and has led to modelling efforts to link the cycling and bioavailability of Fe to atmospheric draw-down of CO_2_ into the ocean [8], [9]. More fundamental research into the biochemical basis of long-term physiological acclimation used by diatoms to survive in low Fe environments provides researchers with more accurate information with which to better model global ocean biogeochemistry.

Over the last two decades, experimental studies to better understand the role of Fe in phytoplankton physiology have used a wide range of approaches from elemental [10] and biophysical analyses [11], [12] to “omics” [13], [14]. A number of cellular strategies have been identified for diatoms residing in Fe-sufficient waters. For example, LaRoche et al. [15] reported that diatoms had significantly higher ratios of the Fe-S protein ferredoxin relative to the non-ferrous flavodoxin. Recently, Whitney et al. [16] demonstrated that the expression of these proteins is controlled by a diel periodicity. In Fe-sufficient waters, Marchetti et al. [10] revealed that open ocean pennate diatoms possess the ability to capitalise on such high Fe conditions by storing excess Fe using the protein ferritin, yet centric diatoms in such offshore waters do not appear to have this protein. In contrast, open ocean centric diatoms survive with extremely low cellular Fe requirements by parsimonious modifications to their photosynthetic architecture [17].

In the last decade, several investigators have utilised two diatom genomes (*T. pseudonana* and *Phaeodactylum tricornutum*) to examine gene expression under Fe-limiting conditions for a range of physiological processes including cell-death [13], Fe acquisition [18], N assimilation [19] and silica bioprocessing [20] in Fe-limited cultures. Transcriptomic studies can provide a better understanding of how diatoms respond to rapid (i.e. quasi-instantaneous) Fe additions or gradual (i.e. days to weeks) decreases in dissolved Fe concentrations due to drawdown by phytoplankton (see Table 1). The ease of real-time PCR gene expression, a useful discovery tool for field-based molecular indicators, is also providing information on individual gene expression across a range of Fe culture conditions [16], [18], [19].

**Table 1:** Methodological details from some recent illustrative studies examining “omics” of Fe limitation on diatoms revealing the wide range of methodological approaches that have been employed.

Study	Protocols	Locale or culture studied	Manipulation	Growth stage	Number of proteins identified	Number of transcripts identified
This Study	Proteomic profiling	*T. pseudonana* CCMP1335	Acclimated to Fe-replete and steady-state Fe limitation	Harvested at mid- exponential growth phase	1850	N/A
Lommer et al. 2012 [46]	Transcriptomics; qRT-PCR; 2D SDS PAGE LC/MS/MS	*T. oceanica* CCMP1005	Fe-replete and Fe stressed cultures	Harvested at late exponential growth phase	767	Chloroplast reads: 2026 (−Fe), 14931(+Fe); Mitochondrial reads: 31261(−Fe), 18136(+Fe).
Durkin et al. 2012 [48]	Transcriptomics	*Pseudo-nitzschia multiseries* CLN 17	Field acclimated; Before and after Fe enrichment	Mid-exponential and stationary (nutrient limited) growth phases	N/A	Using 454-sequencing: Fe-limited surface @ Sta. P: 26; Fe (+) surface @ Sta. P: 37; Puget Sound Surface: 37. SOLiD sequencing: 0 to 375 silicic acid transporter sequence reads detected for each 454-derived SIT sequence.
Marchetti et al. 2012 [14]	Meta-transcriptomics	Field samples from low Fe waters of the northeast subarctic Pacific Ocean	Field acclimated; Before and after Fe enrichment	Line P, northeast subarctic Pacific Ocean; took samples when Fe-limited and after 98 hr Fe addition incubation	N/A	Transcripts for 2845 genes were differentially expressed in +Fe vs. ambient; 3888 in +Fe vs. control.
Whitney et al. 2011 [16]	qRT-PCR; gene expression	*T. pseudonana* CCMP1335, *T. weissflogii* CCMP1010	Acclimated to Fe-deplete and Fe-replete, some with copper limitation	Fe-limiting acclimation or rapid Fe stress (cultures from Fe-replete media were transferred to media with no added Fe)	N/A	No value provided for numbers of transcripts.
Allen et al. 2011 [19]	PCR and Western blots	*Phaeodactylum tricornutum* CCMP2561	Acclimated to Fe-replete and Fe-deplete with varied N sources		212	228 contigs were identified as differentially up-regulated in Fe-limited treatment – these were assembled to 212 predicted proteins.
Mock et al. 2008 [58]	Transcriptomics and proteomics	*T. pseudonana* CCMP1335	Nutrient replete, Si and Fe stressed cultures	Early-stationary growth phase	349	Transcripts for >8000 predicted genes; 75–84 genes induced by various conditions
Kustka et al. 2007 [18]	qRT-PCR	*T. pseudonana* CCMP1335 *Phaeodactylum tricornutum* CCMP630	Acclimated to Fe limitation; Fe-resupply	Steady-state mid-exponential growth phase	N/A	The abundance of specific gene transcripts relative to a housekeeping gene was reported; no values provided.

qRT-PCR = quantitative reverse transcription polymerase chain reaction. 2D SDS PAGE LC/MS/MS = 2 dimensional SDS PAGE gel electrophoresis followed by tandem mass spectrometry protein identifications on individual gel spots.

Recent trends in “omic” examinations of physiological responses of diatoms have focussed on longer-term acclimation responses to a given environmental condition. For example, recent studies have demonstrated that, given sufficient time (conventionally 10 generations) [21] diatoms and other organisms acclimate to conditions of decreasing nutrient supply that are distinct from short-term stress responses [16], [22]. As more “omic” approaches are applied to diatoms, correlations and causations between transcriptomics, proteomics, and metabolomics can be better scrutinized. Since proteomics identifies those proteins that were made and expressed in the cell at the time of harvest, profiling this signature can provide identities of enzymes utilized in various metabolic pathways following acclimation. Although many diatom studies thus far have quantitatively examined gene expression, none have taken advantage of recent developments in proteomic profiling that reveal low abundance proteins, thereby providing the full complement of proteins expressed and possibly a new viewpoint of the effects of acclimation on the proteome.

The aim of our study was to identify, using the proteome, physiological strategies (during mid-exponential growth) of diatoms exposed to long-term acclimation to high or low Fe conditions. We thus examined the proteome of cultures acclimated to specific Fe conditions for 10 generations. Proteomics can be used to decipher biochemical pathways [23], [24] and these pathways are likely to be altered depending on whether diatoms encounter quasi-instantaneous or gradual changes in Fe supply. In our study we used a data-independent acquisition strategy of shotgun proteomic profiling [25]–[27] to survey the dynamic diatom proteome and map the biochemical pathways utilized by the coastal diatom *T. pseudonana* to survive under low Fe conditions.

## Methods

### Acclimation of *T. pseudonana* culture

The diatom *T. pseudonana* was selected as the study organism as it has a well characterized physiology, genome, and proteome [28]–[31]. *T. pseudonana* clone 3H (CCMP1335, mean diameter 4 µm) was obtained from the Provasoli-Guillard Center for Culture of Marine Phytoplankton (West Boothbay Harbor, ME, USA). Cells were grown axenically using semi-continuous batch culturing and the chemically well-defined artificial seawater medium AQUIL [32], [33]. The cultures were grown in 28 mL polycarbonate tubes and were acclimated to either Fe-limiting (pFe 20.5, where pFe = log [Fe^3+^] and [Fe]_total_ = 42 nmol L^−1^) or Fe sufficient conditions (pFe 19, [Fe]_total_ = 1.37 µmol L^−1^), with speciation calculated using MINEQL [34]. The AQUIL medium used in this study included 100 µmol L^−1^ EDTA to buffer the trace metals, and was prepared with a chemical composition identical to that described by Maldonado et al. [35]. All cultures were maintained at 19±1°C and a continuous light intensity of 150 µmol quanta m^−2^ s^−1^, provided with cool-white fluorescent lights. The growth rates (d^−1^) of the cultures were monitored daily using in vivo chlorophyll a (Chl) fluorescence measurements with a Turner Designs AU-10 Fluorometer (Sunnyvale, CA, USA). Cell density (cells mL^−1^) and cell volume (fL cell^−1^; fL = femtoliter = 10^−15^ L) were determined on freshly harvested cells using a Coulter Z2 Particle Count and Size Analyzer. Sterile trace metal-clean techniques were used during all experiments and manipulations.

A Phyto-PAM fluorometer (Walz) equipped with a Phyto-ED emitter-detector unit was used to measure the maximum photochemical efficiency of photosystem II (F_v_/F_m_). All fluorescence measurements were made once algal samples had been dark-acclimated for 30–45 minutes at 19±1°C. The fluorometer was programmed to acquire multiple turnover saturations of PSII using a 200–300 ms saturation flash applied at 30 s intervals. The inter-pulse interval was determined from the minimum time required for fluorescence to relax to pre-saturation levels. The primary signals (F_o_ and F_m_) measured by the Phyto-PAM were obtained by excitation of the sample with measuring light beams at 4 different wavelengths (470, 520, 645 and 665 nm). The mean value of these 4 signals was calculated for each saturation flash, and a minimum of 10 saturation flashes was averaged for each sample. A Water-S stirring device (Walz) was used to keep samples suspended, and was shut off 10 seconds before applying each saturation flash to minimize signal noise. Differences in growth rates, F_v_/F_m_, and cell volumes between Fe treatments were assessed using a two-tailed T-test (p<0.05) following Levene's Test for homoscedasticity.

Once the cells were acclimated to the specific Fe levels (growth rates in 10 successive transfers varied by less than 15%, Brand et al. 1981), they were used to inoculate 1 L cultures in polycarbonate bottles. These large volume cultures were monitored daily by measuring cell density and volume, as well as Chl fluorescence. At mid-exponential phase, cells were harvested by filtration onto 47 mm diameter polycarbonate filters, resuspended in a small volume of media and pelleted by centrifugation (4°C, 7,500× g, 5 min) into four separate eppendorf tubes. The pellets were frozen in liquid nitrogen and stored at −80°C.

### Proteomic sample preparation

Details of cellular preparations for proteomic analyses can be found in Nunn et al. [36]. Briefly, 6 M urea was added to the cell pellets and they were lysed using a titanium micro-probe sonicator. Between each sonication event of 10–15 s, samples were immersed in liquid nitrogen to rapidly cool them down. After 10 sonication events, disulfide bonds were reduced with dithiothreitol and alkylated with iodoacetamide. Ammonium bicarbonate was added to dilute the urea prior to the addition of MeOH. The combination of urea, a strong denaturing agent, and methanol helped to solubilize membrane proteins. No cellular or chemical fractionations were conducted prior to Trypsin digestions in order to reduce protein loss. Each sample received Trypsin at an enzyme∶protein ratio of 1∶50, vortexed, and incubated on a Thermomixer (800 rpm) overnight at 37°C. Samples were desalted using a macro-spin C18 column (NestGroup) following the manufacturers guidelines prior to analysis by mass spectrometry (MS). Peptide concentrations were measured on each sample using the Thermo Scientific NanoDrop 2000/2000c Spectrophotometer. The peptide bond absorbance was monitored at 205 nm UV wavelength and samples were diluted to yield a final concentration of 100 µg protein ml^−1^.

### Mass Spectrometry

Samples were separated and introduced into the mass spectrometer (MS) by reverse-phase chromatography using an 15 cm long, 75 µm i.d. fused silica capillary column packed with C18 particles (Magic C18AQ, 100 Å, 5 µ; Michrom, Bioresources, Inc., CA) fitted with a 2 cm long, 100 µm i.d. precolumn (Magic C18AQ, 200 Å, 5 µ; Michrom). Peptides were eluted using an acidified (formic acid, 0.1% v/v) water-acetonitrile gradient (5–35% acetonitrile in 60 min). Mass spectrometry was performed on two Thermo Fisher (San Jose, CA) hybrid tandem mass spectrometers: the linear ion trap Velos (LTQ-VELOS) and the linear ion trap –Orbitrap (LTQ-OT). Based on peptide concentrations, a total of 1 µg of peptide digest in 10 µl of 5% ACN, 0.1% formic acid was sampled per LC-MS analysis. For quantitative analyses, the 4 biological splits from Fe-limited (Tp_1_^−^, Tp_2_^−^, Tp_3_^−^, Tp_4_^−^) and Fe-replete(Tp_1_^+^, Tp_2_^+^, Tp_3_^+^, Tp_4_^+^) diatoms were analysed on the LTQ-OT with four gas phase fractionations using data-dependent acquisition (DDA), as is outlined in Nunn et al. [36], culminating in a total of 16 analyses per condition. As previously mentioned, no chemical or cellular fractions were completed on the whole cell lysates. Gas phase fractionations in the mass spectrometer provide the user with an accurate means for isolating peptides based on the *m/z* ratio with no sample loss. Gas phase fractions (GPF) selected were optimized using the *Thalassiosira pseudonana* genome to predict the best *m/z* windows (350–444, 444–583, 583–825, 825–1600 *m/z*) [37]. This set of quadruplicate GPF analyses on the LTQ-OT provided a dataset from which statistical confidence could be applied to determine significantly up- or down-regulated proteins with respect to the alternate cell state using *QSpec* (see below).

In addition, the Fe-replete (Tp_1_^+^) and Fe-limited (Tp_1_^−^) cell lysates were analyzed on the LTQ-VELOS using a data-independent analysis (DIA) known as the PAcIFIC method [27]. This method is ideal for examinations of low abundance proteins and yields a rapid automated profile of all proteins expressed at the time of harvest (down to an estimated copy number of 100 proteins per cell) [27]. This dataset provided the means to interrogate the presence or absence of full metabolic pathways. Rather than requiring the mass spectrometer to select ions for fragmentation based on its observation in the MS1 scan, it is programmed to fragment all ions in a narrow range of *m/z* units. Slight modifications to the published PAcIFIC method were made to adapt the protocol to the LTQ-VELOS' faster duty cycle. Each method file included a 60 min linear HPLC gradient of 5–35% ACN and the MS covered a 31.5 *m/z* mass range, rather than 15 *m/z* units. This resulted in a total of 32 method files per PAcIFIC analytical cycle, covering a full *m/z* range of 400–1400 *m/z*. Triplicate PAcIFIC analytical cycles were completed on both the Fe-replete (Tp_1_^+^) and Fe-limited (Tp_1_^−^) samples, yielding a total of 96 MS experiments.

Confirmation of the up- or down-regulation of a pathway was confirmed using the quadruplicate, statistically rigorous, LTQ-OT dataset. The PAcIFIC dataset provides information on the presence or absence of a larger number of proteins and can provide spectra from low abundant proteins, but the quadruplicate sets of 4 GPF analyses provided data for spectral counting with statistical confidence.

### Protein Database Searching and Mass Spectrometry Data Interpretation

All tandem mass spectrometry results were searched and interpreted with an in-house copy of SEQUEST (PVM v.27 20070905) following the parameters outlined in Nunn et al. [31]. The protein database used for correlating spectra with protein identifications was generated by combining the latest release version 3.0 of the nuclear *T. pseudonana* predicted protein database (www.jgi.doe.gov), the unmapped sequences (Thaps3_bd; www.jgi.gov), 302 *T. pseudonana* proteins from the PubMed Entrenz Protein database, and 50 common contaminants. SEQUEST parameters included: reverse concatenated sequence database search, no enzyme specificity, cysteine modification of 57 Da (resulting from the iodoacetamide), and modifications on methionine of 15.999 Da (oxidation). Minimum protein and peptide thresholds were set at p>0.95 on ProteinProphet and PeptideProphet [38]. The SEQUEST criteria for a doubly charged peptide used a correlation factor (Xcorr) >2.5, a cross-correlation factor ΔCorr>0.1 and for a triply charged peptides the Xcorr minimum was 3.5. Protein identifications from the whole-cell lysates were accepted by ProteinProphet if the above mentioned thresholds were passed, two or more peptides were identified (PeptideProphet), and at least one termini was tryptic. Using concatenated target-decoy database searches, false-discovery rates (FDR) were calculated according to Elias and Gygi [39] and were all <1%.

### Label-free protein Quantification (QSpec)

For protein quantification, 4 biological splits were collected from both the Fe-limited and Fe-replete cultures. Each biological split received four 90-minute gas phase fraction analyses on the LTQ-OT, resulting in 16 tandem MS experiments per culture condition. This dataset was used in order to determine relative quantities of protein expression from the Fe-replete and Fe-deplete cultures. The common method of spectral counting was employed to determine relative quantities [40]–[42]. Spectral counting sums up the number of identified peptide tandem mass spectra resulting from a specific protein in order to estimate abundance of that protein relative to other proteins in the sample [43]. Final spectral counts of each protein result from the accumulation of all spectral counts derived from all 4 GPF analyses per analytical set. Spectral counting data were filtered at p>0.95 protein and peptide probability using PeptideProphet; proteins with only one peptide identified were excluded. Significance analyses were completed using *QSpec* in order to determine if proteins were significantly up- or down-regulated based on the quadruplicate sets of 4 GPF analyses. *QSpec* was designed specifically for interpreting differences in protein populations determined from tandem mass spectrometry spectral counts [42]. *QSpec* takes into account the size of the protein and normalizes total spectral counts achieved per analytical set if needed. *QSpec* is reported using a fold change difference in abundance with a log base 2 scale. This provides an easy way to examine the data because a reported positive fold change indicates up-regulation in Fe-limited cells (e.g. +1 fold means twice, or 2^1^, as many spectra were observed in the Fe-limited relative to the Fe-replete cells), and a negative fold change indicates down-regulation of a protein in Fe-limited cells. A reported fold change of zero indicates no significant difference was measured between the quadruplicate data sets from Fe-limited and Fe-replete diatoms. Proteins were considered to be up- or down- regulated if the reported Bayes Factor was >10, the corresponding FDR<1%, and the fold difference observed was >0.5.

### Ipath: metabolic pathway mapping

To better illustrate the potential physiological implications of the changes to the *T. pseudonana* proteome, we mapped expressed proteins in the context of overall metabolic pathways using iPath software (http://pathways.embl.de) [44], [45]. Each protein's observed presence was determined from the LTQ-VELOS-generated PAcIFIC data. Approximately 50% of the proteins in the *T. pseudonana* proteome have iPath identifiers, such as an enzyme commission numbers (EC numbers), or associated KEGG pathways (Kyoto Encyclopaedia of Genes and Genomes) and can be mapped using iPath software.

## Results

Iron-limited cells grew significantly slower (0.68±0.26 d^−1^; mean ± standard error) than Fe-replete cells (1.71±0.25 d^−1^) (p<0.05, two-tailed t-test; Table 2). The photochemical efficiency of PSII, F_v_/F_m_, was lower in Fe-limited cells (0.54±0.01) compared to Fe-replete cells (0.67±0.01; Table 2). Fe-limited cells of *T. pseudonana* had significantly lower specific growth rates and F_v_/F_m_, and were significantly smaller, than Fe-replete cells (p<0.05) (Table 2).

**Table 2:** Specific growth rates (d^−1^), photochemical efficiency of photosystem II (F_v_/F_m_), and cell volumes of Fe-replete and Fe-limited cultures of the diatom *T. pseudonana* CCMP1335 used for proteomic analyses.

Treatment	Specific growth rate (d^−1^)	F_v_/F_m_	Cell volume (fL cell^−1^)
Fe-replete	1.71±0.25	0.67±0.01	5.47±0.14
Fe-limited	0.68±0.26	0.54±0.01	3.93±0.08

Values are mean ± standard error. fL = femtoliter = 10^−15^ liter.

**Figure 1:**
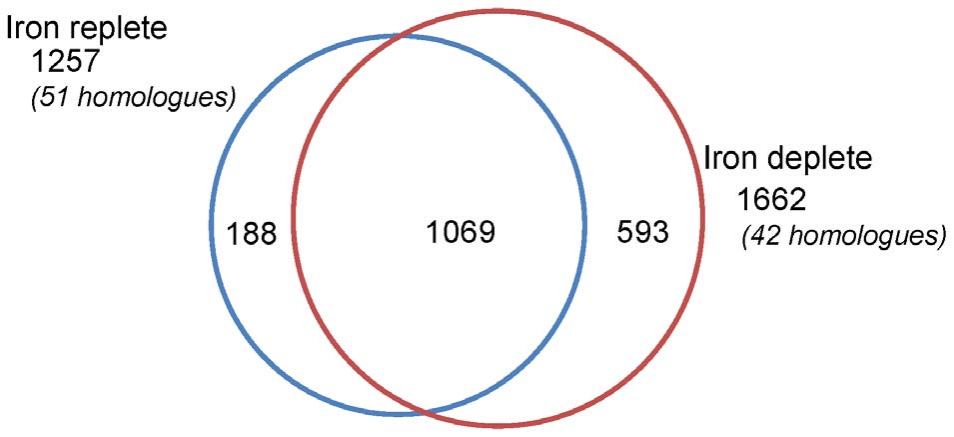
Venn diagram of number of proteins identified in Fe-replete and Fe-limited *T. pseudonana*. Proteins results presented were confidently identified from triplicate PAcIFIC analyses on the LTQ-VELOS. Fe-replete (blue) and Fe-limited (red) conditions were harvested at mid-exponential growth phase after acclimation. Numbers parenthetically annotated indicate homologous protein identifications.

A total of 1850 proteins (containing 2 or more peptides) were identified from the combined Fe-limited and Fe-replete datasets, providing the largest expressed protein dataset of diatoms to date (Table S1). Of the 1257 identifications from Fe- replete cells, 85% overlapped with the Fe-limited diatoms, revealing 188 and 593 proteins uniquely expressed under Fe-replete and Fe-limiting conditions, respectively (Figure 1).

Our GPF dataset revealed 131 proteins that were significantly up- or down-regulated (*QSpec*; threshold of >10 Bayes factor). *QSpec* identified 77 proteins to be up-regulated in Fe-limited cells, and 54 to be up-regulated in Fe-replete cells, by more than a 0.5 fold change in log_2_ scale (i.e. 0 fold change indicates no difference in abundance was measured; Table S2). Unlike transcript regulation, quadruplicate datasets of protein expression with >0.5 fold difference is considered a significant difference in protein abundance [42], [46]. Gene ontology categories represented by the up-regulated proteins from each cell culture condition in Table S2 were analyzed using DAVID software to identify which metabolic pathways had a significant number of proteins up-regulated and were associated with a specific physiological process and/or biochemical pathway (Table 3 and 4). MS analyses on Fe-replete cells indicate that 20 of the proteins up-regulated (*QSpec*-analysis) were involved in translation and 19 were involved in photosynthesis and light harvesting (Table 3). Other well-represented biochemical categories in Fe-replete cells included ion transporters, macromolecular biosynthetic processes, and proteins involved in aspects of genetic information and processing other than translation. Although Fe-limited cells up-regulated more proteins, the metabolic pathways associated with these proteins were more diverse, resulting in fewer, well-represented pathways. When acclimated to Fe-limited conditions, *T. pseudonana* up-regulated proteins involved in the metabolic processing of sugars, amino acid metabolism, and protein transport and localization (Table 4).

**Table 3:** Biological processes up-regulated in Fe-replete *T. pseudonana* as reported by DAVID Biological Process Term level 4 analysis of gene ontology categories.

*Biological Process Term*	*Count*	*%*	*P-Value*
translation	20	30.8	1.2E-09
generation of precursor metabolites and energy	15	23.1	1.2E-07
photosynthesis	11	16.9	2.1E-06
photosynthesis, light reaction	8	12.3	1.8E-05
proton transport	7	10.8	3.2E-05
hydrogen transport	7	10.8	3.2E-05
ATP biosynthetic process	7	10.8	1.7E-04
ATP metabolic process	7	10.8	1.7E-04
energy coupled proton transport, down electrochem. gradient	6	9.2	2.3E-04
ATP synthesis coupled proton transport	6	9.2	2.3E-04
ion transmembrane transport	6	9.2	2.6E-04
purine nucleoside triphosphate biosynthetic process	7	10.8	2.7E-04
purine ribonucleoside triphosphate metabolic process	7	10.8	2.7E-04
ribonucleoside triphosphate biosynthetic process	7	10.8	2.7E-04
purine nucleoside triphosphate metabolic process	7	10.8	2.7E-04
nucleoside triphosphate biosynthetic process	7	10.8	2.7E-04
ribonucleoside triphosphate metabolic process	7	10.8	2.7E-04
purine ribonucleoside triphosphate biosynthetic process	7	10.8	2.7E-04
nucleoside triphosphate metabolic process	7	10.8	2.9E-04
plasma membrane ATP synthesis coupled proton transport	3	4.6	6.8E-04
oxidative phosphorylation	6	9.2	7.0E-04

Count: total proteins up-regulated in Fe-replete cultures that correlated with the Biological Process; %: Percent of total proteins associated with that term; P-value: probability that the number of proteins identified to be up-regulated from that biological process is significant with respect to the total number of proteins from the *T. pseudonana* proteome associated with that process (reported p-value threshold <0.05).

**Table 4:** Biological processes up-regulated in Fe-limited *T. pseudonana* as reported by DAVID Biological Process Term level 4 analysis of gene ontology categories.

*Biological Process Term*	*Count*	*%*	*P-Value*
hexose metabolic process	6	7.8	1.1E-03
monosaccharide metabolic process	6	7.8	1.2E-03
glucose metabolic process	5	6.5	4.7E-03
glycolysis	4	5.2	6.8E-03
aspartate family amino acid biosynthetic process	3	3.9	1.2E-02
hexose catabolic process	4	5.2	1.4E-02
glucose catabolic process	4	5.2	1.4E-02
monosaccharide catabolic process	4	5.2	1.4E-02
aspartate family amino acid metabolic process	3	3.9	1.7E-02
cellular carbohydrate catabolic process	4	5.2	2.0E-02
alcohol catabolic process	4	5.2	2.0E-02
cellular metabolic compound salvage	2	2.6	3.6E-02
asparagine metabolic process	2	2.6	3.6E-02
asparagine biosynthetic process	2	2.6	3.6E-02
establishment of protein localization	5	6.5	3.9E-02
protein transport	5	6.5	3.9E-02
protein localization	5	6.5	4.6E-02
intracellular protein transport	4	5.2	4.9E-02
generation of precursor metabolites and energy	6	7.8	5.2E-02
cellular protein localization	4	5.2	5.6E-02
cellular macromolecule localization	4	5.2	5.6E-02
carbohydrate catabolic process	4	5.2	6.2E-02
intracellular transport	4	5.2	7.6E-02

Count: total proteins up-regulated in Fe-limited cultures that correlated with the Biological Process; %: Percent of total proteins associated with that term; P-value: probability that the number of proteins identified to be up-regulated from that biological process is significant with respect to the total number of proteins from the *T. pseudonana* proteome associated with that process (reported p-value threshold <0.05).

**Figure 2:**
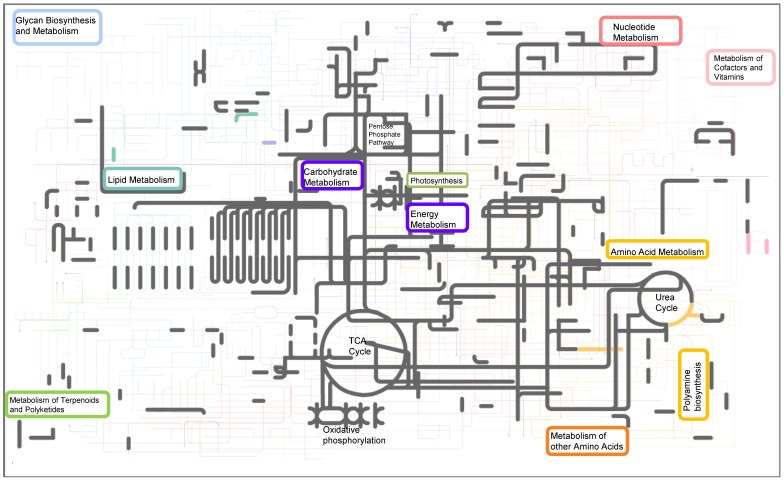
Metabolic biochemistry map of proteins expressed and identified in Fe-replete *T. pseudonana*. Map includes data from triplicate PAcIFIC analyses on a tandem mass spectrometer from *Thalassiosira pseudonana* acclimated to Fe-replete conditions. Each node (or corner) represents a metabolite and the lines connecting the nodes represent an enzyme (i.e. protein). Metabolites were not measured in this study. Proteins that were identified in both Fe-replete and Fe-limited cultures are highlighted in grey. Proteins that were identified to be unique to the Fe-replete cultures are indicated in color. From top left – light blue: sugar and glycan biosynthesis, light purple: starch and sucrose metabolism (including photosynthesis, oxidative phosphorylation, carbon fixation), dark purple: glycolysis-gluconeogenesis (including TCA cycle), red: nucleotide metabolism, teal: lipid metabolism, orange: amino acid metabolism (including urea cycle).

**Figure 3:**
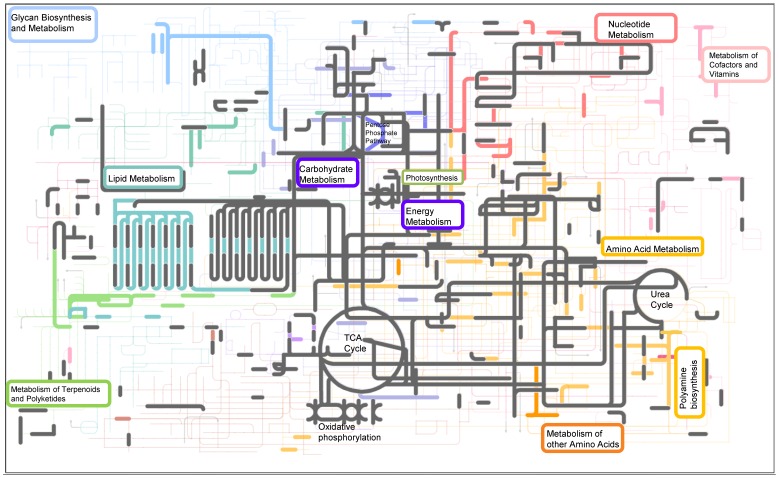
Metabolic biochemistry map of proteins expressed and identified in Fe-limited *T. pseudonana*. Map includes data from triplicate PAcIFIC analyses on a tandem mass spectrometer from *Thalassiosira pseudonana* acclimated to Fe-limitation. Each node (or corner) represents a metabolite and the lines connecting the nodes represent an enzyme (i.e. protein). Metabolites were not measured in this study. Proteins that were identified in both Fe-replete and Fe-limited cultures are highlighted in grey. Proteins that were identified to be unique to Fe-limited cultures are indicated in color. From top left – light blue: sugar and glycan biosynthesis, light purple: starch and sucrose metabolism (including photosynthesis, oxidative phosphorylation, carbon fixation), dark purple: glycolysis-gluconeogenesis (including TCA cycle), red: nucleotide metabolism, teal: lipid metabolism, orange: amino acid metabolism (including urea cycle).

Characterization of metabolic pathways was improved by identifying low abundance proteins using PAcIFIC mass spectrometry (Figure 2, 3). Twice as many protein identifications from the lipopolysacharide biosynthesis pathway were identified in the Fe-limited cultures (14 proteins) compared to the Fe- replete cultures (7 proteins; Table S3). A few of the proteins in lipopolysacharide biosynthesis pathway were also significantly up-regulated in Fe-limited cultures (UDP-glucose 6-dehydrogenase, +1.8 fold and UDP-glucose pyrophosphorylase, +1.23 fold; Table S2). Other critical enzymes in the pathway were uniquely identified in Fe-limited cells, such as UDP-sugar pyrophosphorylase UDP-glucose 4 epimerase. The pentose phosphate pathway was also better represented (twice as many proteins) in the Fe-limited compared to Fe-replete cultures (Table S4, Figure S1A and S1B). Transketolase, an enzyme involved in pentose phosphate pathway and C fixation, was up-regulated ∼2-fold in Fe-limited cells, supporting the pathway analysis (Table S2, S4 and Figure 3). The rate-controlling enzyme for the pentose phosphate pathway, glucose-6-phosphate dehydrogenase, was only confidently identified in Fe-limited cells. Many of the metabolites resulting from the pentose phosphate pathway enter glycolysis, and although all the glycolysis enzymes are constitutively expressed in Fe-replete and Fe-limited cultures, several of the key enzymes were up-regulated in the Fe-limited cultures (Table S2).

The eight glycolysis-specific proteins that are up-regulated include fructose-1,6-bisphosphate aldolase precursor (+1.78 fold), glucose-6-phosphate isomerase (+1.75 fold), fructose bisphosphate aldolase (+1.65 fold), phosphofructokinase (PFK-1;+1.50 fold), enolase (+1.37 fold), fructose-1,6-bisphosphate aldolase precursor (+0.65 fold), glyceraldehydes-3-phosphate dehydrogenase precursor (GADPH; +0.6 fold), and phosphoglycerate kinase precursor (+0.5 fold) (Table S2 and S5).

In the Fe-limited cultures, 35 more proteins from the amino acid biosynthesis and degradation pathways were identified (total of 112 proteins; Figure 3 and Table S6). In particular, 15 proteins involved in the biosynthesis of phenylalanine, tyrosine, and tryptophan were identified in Fe-limited cultures, whereas only 5 enzymes involved this pathway were identified in the Fe-replete cultures. PAcIFIC analyses identified two aminotransferases unique to the Fe-limited cultures, and two additional aminotransferases were also statistically determined to be up-regulated in the GPF dataset (Table S2). Many proteins involved in amino acid metabolism are also associated with N metabolism (Table 5). Nitrate and nitrite transporters were identified in both cell states, in addition to urea transporters and the urease enzyme (Table 5). The Ni-ABC transporter was, however, the most down-regulated protein in Fe-limited cells in this study (−2.87 fold; Table S2). Ni-ABC transporters utilize the ATP-binding cassette to help energetically transport Ni across membranes. The nickel transported into the cell is an essential co-factor for the urease enzyme. NADPH nitrite reductase was confidently identified in both cell states (20 unique peptides identified in each cell state), whereas the ferredoxin nitrite reductase was only identified in the Fe-limited cultures (6 unique peptides in Fe-limited diatoms only). In the case of all of other N metabolic enzymes, a greater number of unique peptides were identified in all analyses of the Fe-replete cultures.

**Table 5:** Spectral count data and QSpec statistical analyses for key nitrogen metabolism, urea cycle, and spermine synthesis proteins identified in all experiments.

		Fe replete					Fe limited					QSpec Statistics		
Global ID Number	Protein Function	4GPFS/replicate				Triplicates on VELOS	4GPFS/replicate				Triplicates on VELOS	Bayes factor	Log_2_ fold change	Significantly up/down regulated
	Replicate#	*Tp_1_^+^*	*Tp_2_^+^*	*Tp_3_^+^*	*Tp_4_^+^*	*PAcIFIC Tp_1_^+^*	*Tp_1_^−^*	*Tp_2_^−^*	*Tp_3_^−^*	*Tp_4_^−^*	*PAcIFIC Tp_1_^−^*			
**224013245**	glutamine synthase	78	75	76	80	69	44	53	44	52	57	112.00	0.46	
**223999327**	carbamoyl-phosphate synthetase III; CPSase III	19	20	27	28	36	23	21	21	23	37	0.52	0.07	
**224009263**	glutamate synthase	5	7	6	8	19	4	4	3	5	26	1.75	0.42	
**224011010**	ferredoxin-dependent glutamate synthase	4	2	2	4	14	6	4	5	4	21	1.20	0.41	
**209583549**	argininosuccinate synthase	6	5	6	6	10	11	13	7	8	20	3.37	0.48	
**223995983**	nitrite reductase (NAD(P)H) large subunit	13	16	10	12	21	15	17	10	13	20	0.56	0.06	
**224009908**	**aspartate-ammonia ligase**	**1**	**2**	**0**	**0**	**12**	**5**	**8**	**8**	**6**	**19**	**40.64**	**1.63**	**YES**
**224013082**	**N-acetylornithine aminotransferase**	**2**	**1**	**4**	**2**	**7**	**6**	**8**	**6**	**7**	**16**	**28.15**	**0.91**	**YES**
**223993805**	OTC: ornithine transcarbamylase	4	4	3	3	10	3	2	2	3	14	1.03	0.25	
**224000259**	oat-prov protein-ornithine aminotransferase	5	8	8	8	10	11	8	6	7	14	0.62	0.09	
**223996511**	**spermine synthase**	**5**	**11**	**6**	**8**	**5**	**19**	**16**	**11**	**15**	**14**	**43.36**	**0.66**	**YES**
**223999447**	**asparagine synthetase**	**0**	**0**	**0**	**0**	**4**	**2**	**3**	**4**	**6**	**12**	**10.25**	**1.63**	**YES**
**224010906**	nitrate reductase	2	2	1	1	14	0	0	0	1	11	1.09	0.84	
**224014054**	hydrolase/nickel ion binding/urease	2	2	2	2	7	1	1	1	1	11	1.15	0.44	
**224001546**	spermidine/putrescine ABC transporter	2	3	2	4	7	2	3	2	5	11	0.59	0.06	
**223999105**	glutamate dehydrogenase	1	1	3	3	5	2	1	1	2	10	0.71	0.20	
**224005475**	argininosuccinate lyase	0	0	0	0	3	1	1	0	1	8	1.46	0.77	
**223993093**	ornithine cyclodeaminase	2	0	0	0	9	2	1	2	0	8	0.71	0.48	
**224012585**	glutamine synthetase	0	0	0	0	3	2	3	2	2	7	3.03	1.34	
**224003823**	NADH glutamate synthase small chain	2	0	1	1	8	3	3	1	1	7	0.93	0.42	
**224007933**	solute∶sodium symporter/urea transporter	7	5	6	5	11	4	4	5	5	7	0.77	0.22	
**223997254**	nitrite transporter NAR1	2	3	4	3	8	1	2	1	1	7	2.53	0.66	
**223999185**	ferredoxin nitrite reductase	0	0	0	0	0	4	4	1	0	6	4.56	1.34	
**223995861**	carbamate kinase	0	0	0	0	0	0	1	1	0	6	1.40	0.58	
**224006530**	mitochondrial glycine decarboxylase T-protein	2	1	1	1	0	2	3	3	3	5	1.71	0.57	
**223998258**	putative nitrate transporter	2	0	1	1	11	0	0	1	0	5	1.08	0.62	
**209585936**	nitrate transporter	2	2	4	5	4	2	4	3	3	4	0.72	0.05	
**224008166**	aminomethyltransferase	0	0	0	0	2	0	0	0	0	2	1.00	0.00	
**224008464**	nitrate reductase apoenzyme	0	0	0	0	0	0	0	1	0	2	0.99	0.34	
**209586149**	arginase	0	1	2	1	2	0	0	0	0	0	1.00	0.00	
**224014840**	putative leucine dehydrogenase	0	0	0	0	0	0	0	0	0	2	Identified only using PAcIFIC		
**223998388**	glycine cleavage protein (aminomethyltransferase)	0	0	0	0	0	0	0	0	0	2	Identified only using PAcIFIC		

Each replicate analysis (i.e. 1, 2, 3, 4) is indicative of the summation of spectral counts for that particular protein identified in 4 gas phase fractions (GPFs). Spectral counts resulting from triplicate analytical cycles of PAcIFIC were added together to provide a final count for the PAcIFIC analysis. In order to be considered significantly up or down regulated two criteria were met by QSpec: Bayes factor >10, and log_2_(−Fe/+Fe)>0.5. Proteins that do not have QSpec information were only identified using the data-independent PAcIFIC method and could not be statistically evaluated. This list does not include all proteins involved in nitrogen metabolism (e.g. amino acids biosynthesis and degradation).

**Figure 4:**
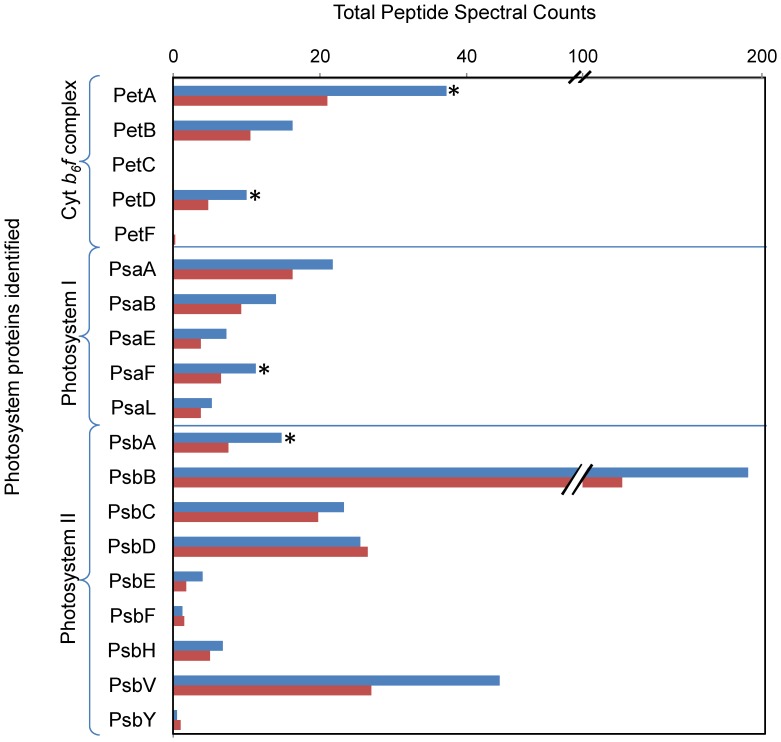
Total peptide spectral counts from photosystem complex subunits. Spectral counts result from quadruplicate analyses on Fe-replete (blue) and Fe-limited (red) cultures. Photosystem II requires 2–3 atoms of Fe per complex, Cytochrome (Cyt) *b_6_f* complex requires 6 Fe atoms per complex, and photosystem I requires 12 Fe atoms per complex. “*” indicates that the protein was determined to be significantly up- or down-regulated by QSpec (i.e. Bayes Factor >10 and log_2_ fold change >0.5).

All proteins involved in light-harvesting antennae, photosynthetic C fixation and ATP synthase were constitutively expressed (Table S7). However, many of those proteins were identified to be down-regulated in the Fe-limited cultures. A total of 9 PSII complex proteins (Psb A, B, C, D, E, F, H, V, Y) and five photosystem I proteins (Psa B, D, E, F, L) were identified in both Fe-replete and Fe-limited conditions. Photosystem proteins that were identified in Fe-limited cultures yielded lower peptide spectral counts in all subunits except Cytochrome *b_559_* (PsbF) and PSII reaction center D2 (PsbD) (Figure 4). Quantitative analyses revealed that 3 subunits of the chloroplast-localized ATP synthase complex (AtpA, AtpE, and AtpG), the Cytochrome *b_6_f* complex (PetA, PetB), photosystem I ferredoxin binding complex (PsaD), and PSII reaction center D1 (PsbA) were all down-regulated (>0.5 fold) in the Fe-limited cultures (Table S2). Many of these proteins have direct Fe requirements (e.g. heme Fe and Fe-S proteins of the Cyt *b_6_f* complex (Pet A, Pet B, Pet C), or are associated with Fe within a complex (e.g. photosystem I reaction center protein PsaD binding ferredoxin, and PSII reaction center protein D1 associated with a non-heme Fe). Nine fucoxanthin chlorophyll *a/c* binding proteins (FCP) were identified in both cultures, one was significantly up-regulated in Fe-limited cultures, and two were significantly down-regulated (Table S2). The FCP that was up-regulated in Fe-limited cultures has been associated with light mediated oxidative stress [46].

All proteins for the uptake and fixation of CO_2_ were present in the diatoms under both Fe conditions (Table S8). The large subunit of RuBisCO, 1,5-bisphosphate carboxylase/oxygenase (RbcL), responsible for catalyzing the first step of C fixation, was observed to be up-regulated in Fe-limited diatom cultures (Table S2). Because RuBisCO can use either CO_2_ or O_2_ as a substrate, many photosynthetic organisms, including diatoms, employ a C concentrating mechanism to increase the catalytic efficiency of RuBisCO toward CO_2_. Phosphoenolpyruvate carboxylase (PEPC2), which utilizes bicarbonate in order to carboxylate phosphoenolpyruvate into oxaloacetate, was down-regulated in Fe-limited cultures (−1.24 fold; Table S2).

## Discussion

The goal of this study was to better understand the physiological changes in whole-cell metabolism of the cosmopolitan diatom *T. pseudonana* when acclimated to Fe limitation by examining the expressed proteome at mid-exponential steady-state growth. Several investigators have reported gene expression and enzyme assay responses to rapid Fe-limitation [16], [47] and Fe-enrichments or resupply (for examples see Table 1) [18]. Whole-cell diatom responses examined thus far have also revealed physiological responses of field samples before and after quasi-instantaneous Fe enrichments using transcriptomics [14], [48]. The relationship between genomics, transcriptomics, proteomics, and metabolic strategies utilized by an organism is currently being scrutinized [49]–[51]. Recent studies of functional RNA in humans have shown that although much of the genome is transcribed, not all of the transcripts are translated into functional proteins [30], [49], [52]. This suggests that pure genomic analysis or transcriptomic tiling arrays may be limited in their ability to estimate cellular processes and that proteomics may provide an additional metric for determining metabolic strategies. Lommer et al. [46] demonstrated that *T. oceanica* had a very high dynamic range of transcript expression and a relatively narrow range of protein expression in response to varying Fe conditions, suggesting that diatoms, like other organisms, may rapidly increase transcript abundances, but not translate them to proteins.

There is also evidence that proteins can be synthesized within seconds [22], [53], therefore sufficient acclimation to the desired environmental perturbation is essential in order to avoid capturing short-term stress responses. For example, a time series study demonstrated the dynamic proteomic response of a bacterial culture to cadmium exposure in soils [22]; within 15 minutes of exposure to cadmium, the culture manipulated the proteome rapidly enough to be detected using MS-based proteomics, revealing shifts in physiology that were not detected using phylogenetic tools. In order to better understand the adaptive strategies of diatoms to Fe limitation, cultures were acclimated to Fe limitation over 10 generations prior to harvesting them for proteomic analyses. The combination of data independent (DIA) mass spectrometry and such acclimation in our study revealed that many proteins previously determined to be absent in Fe-limited cells actually exhibit a low-level constitutive expression. Although most metabolic pathways involved proteins represented in both the Fe-replete and Fe-limited cells, the Fe-limited cells up-regulated enzymes involved in the pentose phosphate pathway and intracellular protein transport and recycling (Figure S1). In contrast, Fe-replete diatoms up-regulated proteins dedicated to cell division, photosynthesis and the production of macromolecules for energy (Figure S2). The procedure to construct an interactive version of iPath Figures S1 and S2 is provided with the raw data in Text S1. While our primary goal was to understand how diatoms acclimate to steady-state Fe limitation, the metabolic pathways identified to be enhanced in Fe-replete diatoms illustrates that Fe-limitation studies inherently include growth limiting metabolic shifts. In order to tease apart generic growth limitation and Fe-limitation, one would need to do a large-scale study examining the effects of multiple limiting nutrients (e.g. light, temperature, and other nutrients), including individual nutrient limitations and a matrix of co-limitations. This was beyond the scope of this study. In the remainder of the Discussion section, we outline seven important cellular ramifications resulting from the acclimation of our model diatom to differing Fe conditions.

### Intracellular conservation of nitrogen in Fe-limited cells

**Figure 5:**
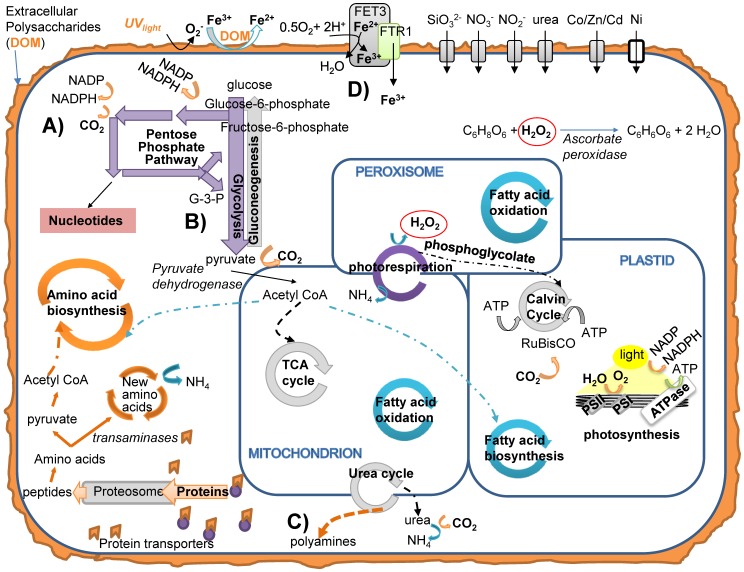
Cartoon representation of diatom cell biochemistry when acclimated to Fe-limitation. Not all metabolic pathways are shown. Black and grey pathways and proteins indicate presence during Fe-limitation, white proteins indicate significantly down-regulated proteins, colored pathways were significantly up-regulated in Fe-limited cells compared to Fe-replete cells. A) Pentose Phosphate Pathway, B) The rejoining of the pentose phosphate pathway with glycolysis and generation of pyruvate. C) Polyamine synthesis using spermine synthase. D) Proposed reduction of Fe^+3^ and eventual transport of Fe into cells. Purple: photosynthesis and glycolysis-gluconeogenesis, red: nucleotide metabolism, teal: lipid metabolism, orange: amino acid metabolism.

Although nitrate is the most abundant N source in the ocean, its use can be constrained by Fe availability as the enzymes involved in nitrate assimilation, nitrate and nitrite reductase, require Fe as a cofactor. Several studies have examined whether Fe-limited diatoms have a preference for, and the ability to utilize, oxidized or reduced forms of N [47], [54], [55]. Regardless of the N source provided to Fe-limited diatoms, the C∶N ratio does not change significantly, suggesting N-uptake enzymes are functioning regardless of the Fe-cofactors required [54]. That said, Milligan et al [46] demonstrated that Fe limited diatoms buid up an internal pool of NO_2_^−^ due to the lack of cellular reducing power present in the cell to adequately use nitrite reductase. Furthermore, nitrite reductase requires 5 Fe atoms per active enzyme, whereas nitrate reductase only requires two. As a result, Fe limited cells have inactive nitrite reductases, resulting in the accumulation of intracellular nitrite and the retardation of nitrate assimilation. Proteomic profiling of the Fe-limited diatom at mid-exponential growth provided evidence of low-level expression of nitrate and nitrite reductases (Table 5). As no ammonium (reduced N source) was added to the culture medium, these findings suggest that Fe-limited diatoms continue to uptake oxidized nitrogen despite a weakened nitrogen assimilation pathway due to the lack of reducing power (as was demonstrated by Milligan et al. [46]). This suggests that oxidized N assimilation is diminished in Fe-limited cells in concert with diminished Fe-limited growth rates. Our proteomic profiles suggest that Fe-limited *T. pseudonana* are primed to compensate for this diminished reducing power and a compromised nitrate assimilation pathway by up-regulating intracellular N recycling pathways. In Fe-limited cells we identified more enzymes involved in intracellular protein trafficking, protein and peptide bond breaking, and the general re-distribution of N and C from protein backbones (Table 4). Evidence for this enzyme-based machinery in Fe-limited cells includes: the unique identification of 14 proteins with proteasome subunits, up-regulation of 2 aminotransferases, the complete pathway for acyl-tRNA biosynthesis, up-regulation of multiple peptidase enzymes, and the identification of 112 proteins involved in amino acid metabolism. Recycling intracellular N-rich proteins would enable the diatom to reduce resources allocated to nitrogen uptake and assimilation (nitrate, nitrite, urea, ammonia), while permitting the cell to regenerate new proteins for growth (e.g. see Figure 5) [19]. Lommer et al. [46] revealed transcriptomic evidence of a similar “biomass recycling” response when *T. oceanica* was subjected to Fe stress and harvested at late-exponential growth phase. This efficient intracellular N recycling strategy may also contribute to the higher diversity of proteins observed in the Fe-limited diatom at mid-exponential growth (Figures 1 and 3).

Fe-limited *T. pseudonana* expressed 35 more enzymes for amino acid metabolism compared to the Fe-replete cells (Figure 3 and Table S6). These included multiple aminotransferases, which can yield many fates for amino acids including re-organization into new amino acids or complete intracellular recycling to alpha keto acids, ammonia, or pyruvate [56]. Argininosuccinate lyase (ArgH), an enzyme that degrades argininosuccinate to arginine and fumerate, was discovered to have a higher protein abundance in the Fe-limited diatoms (Table 5). This suggests the activation of the argininosuccinate shunt during iron limitation and its discovery supports our hypothesis of enhanced intracellular recycling of N-containing compounds. Since arginine can be easily mobilized, degraded, or incorporated into proteins, amino acids, and polyamines (e.g. see Figure 5), it may play a critical role in diatom N recycling and storage [57]. Iron-limited *T. pseudonana* expressed an incomplete urea cycle and up-regulated proteins involved in polyamine synthesis (Table 5 and Figure 2 c.f. 3). The alteration of these biochemical pathways was also noted by Mock et al. [58]. The one enzyme from the urea cycle that we did not detect in Fe-limited cells (Figure 3), arginase, is responsible for the breakdown of arginine into urea. This process appears to primarily occur in Fe-replete cells [36] and in both cell states when they reach stationary growth (data not shown).

Urea can also provide N for diatoms [59]. No significant difference in expression of the urease enzyme was detected between the Fe-replete and Fe-limited cultures. Although previous reports have observed the urease enzyme to be present under Fe and N limitation [19], [47], urease requires nickel (Ni) as a cofactor when processing urea [60], [61]. The Ni-ABC transporter (NikA) was identified to be the most down-regulated protein in Fe-limited cells (−2.87 fold; Table S2). Egleston and Morel [60] demonstrated that the presence of Ni is essential for urease when diatoms are using urea as their primary N source. The down-regulation of the Ni-transporting enzymes suggests that urease was not actively utilized and urea was not a primary N source during Fe limitation. Marchetti et al. [14] conducted Fe-resupply experiments on natural diatom communities that induced the generation of urea cycle transcripts, indicating the pathway's close ties to Fe availability. This tight interlinking of the urea cycle to Fe supply suggests that urea might be a N storage molecule under Fe-replete conditions.

### Glycolysis and the pentose phosphate shunt in Fe-limited cells

Glucose is a primary product resulting from photosynthesis and it can be used in many ways to generate energy in the cell. The up-regulation of the pentose phosphate pathway may allow Fe-limited cells to bypass the first step of glycolysis, which uses ATP, and provide them with excess CO_2_ and reducing equivalents of NADPH (Figure 5A). These reducing substrates may be particularly important in Fe-limited cells by lessening oxidative stress and maintaining cellular integrity. This re-direction of glucose also provides a biochemical route for the cells to create nucleotides from the 5-C molecule ribulose-5-phosphate. The pentose phosphate pathway eventually re-connects with the glycolysis pathway and produces pyruvate, a useful cellular currency (see Figure 5B). In the mitochondria, pyruvate may be converted to acetyl CoA by pyruvate dehydrogenase, which was up-regulated in Fe-limited cells (+1.2 fold). Acetyl-CoA can then either enter the TCA cycle, amino acid biogenesis, or fatty acid biosynthesis – pathways that had the full complement of necessary enzymes identified in Fe-limited diatoms (Figures 3 and 5).

The enzymes involved in glycolysis and the pentose phosphate pathway identified in this study have been previously shown to play a primary role in the conversion of glucose to energy in times of low photosynthetic competence or activity, such as under Fe limitation [62]–[65]. The over-expression of enzymes in the glycolytic pathway by photosynthetic organisms stressed by Fe has been previously noted in green alga [62], tomatoes [63], and in cucumber roots [64], [65]. To our knowledge, this is the first time that the pentose phosphate pathway has been identified to play a key role in the acclimation of diatoms to Fe limitation by providing cells additional reducing equivalents via the pentose phosphate shunt.

### Photosynthetic energy production in Fe-deplete cells

In most photosynthetic organisms, light-driven adenosine triphosphate (ATP) synthesis via photosynthesis is the primary pathway for generating chemical energy that can be used to drive other metabolic pathways. Many of the photosynthetic enzymes were previously identified to be the most abundant in nutrient replete *T. pseudonana* cells [31]. We would expect the down-regulation of photosynthetic proteins when Fe limits diatoms because many require Fe as a cofactor in order to transfer electrons to the ATP synthase complex [55]. Photosystem I proteins and Cytochrome *b_6_f* require Fe and are down-regulated in Fe-limited cultures (Table S7) [11], [17], [46]. This study revealed that *T. pseudonana* acclimated to low Fe conditions retains the core complexes of both PSI and PSII, but most subunits of these complexes are down-regulated in Fe-limited cells (Figure 4). One key function of these photosynthetic complexes is to pump protons across the thylakoid membrane of the chloroplast, which drives the production of ATP by ATP synthase. Under Fe limitation, three chloroplast-localized ATP synthase subunits are down regulated (Table S2).

The presence of both photosystem complexes suggests that they are working collaboratively, but they are generating fewer protons to drive ATP synthase, resulting in fewer ATP synthase complexes being present. This would suggest that the limited Fe in the cell is retained in the PSII and PSI complexes and to control growth, ATP synthase is actively synthesized or recycled, depending on growth status. This can result in a reduction of net energy gained and slower growth rates in Fe-limited diatoms (Table 2).

### Enhancement of photorespiration in Fe-limited cells

Two enzymes responsible for providing substrates for the C-concentrating mechanism (CCM) are carbonic anhydrase and phosphoenolypyruvate carboxylase (PEPC2). The higher abundance of PEPC2 in Fe-replete cells compared to Fe-limited cells suggests that when diatoms are provided excess nutrients they are capable of actively avoiding photorespiration by using CCMs, whereas the low-abundance of PEPC2 in Fe-limited cultures may force them to photorespire [14]. An enhanced role for photorespiration under Fe limitation is supported by the unique identification of two photorespiration enzymes using the LTQ-VELOS PAcIFIC profile (phosphoglycolate phosphatase and glycine decarboxylase). Photorespiration can result in a net loss of C and the increased presence of proteins in this pathway provides a biochemical explanation for the reduced C∶N ratio found in *T. pseudonana* acclimated to Fe limitation [54].

The consequences of Fe-limited diatoms photorespiring, rather than employing a CCM, include net cellular C and N loss, slower growth rates, and the production of ammonia. Ammonia has many potentially useful fates in diatoms including (but not limited to) amino acid generation and polyamine synthesis (Figure 5C). Spermine synthase, a key enzyme in polyamine synthesis, which helps precipitate silica on cell wall frustules, was also identified to be up-regulated in Fe-limited cells (Table 5, Table S2). We propose that photorespiration, through its link with spermine synthase and its substrate ammonia, may underlie the increased Si∶N ratio [66] and the thickened silica frustule observed in Fe-limited *T. pseudonana* [10], [67].

### Iron acquisition and endocytic recycling in Fe-limited cells

We predicted that *T. pseudonana* could increase production of proteins involved in Fe acquisition when limited by Fe. Unlike oceanic pennate diatoms, such as *Pseudo-nitzschia* sp. and *Fragilariopsis* sp., *T. pseudonana* does not have the gene encoding ferritin, a protein used for intracellular Fe storage [10]. The lack of ferritin may contribute to the higher extracellular Fe levels (or demand) for *T. pseudonana* to thrive compared to oceanic diatoms.

Iron permease, FTR1, was up-regulated in the Fe-limited cells (Table S2; +0.77 fold). FTR1 requires the copper-requiring ferroxidase (FET3) to function properly [68], [69]. The FET3 enzyme was not identified in our study, possibly because it is membrane-bound and difficult to extract and ionize [70]. Strochlic et al. [68] observed that yeast cells maintain and recycle the FTR1 complex under Fe limitation, whereas during Fe-replete conditions the cells targeted this complex for endocytic recycling, regenerating the protein when needed. This may explain the observed up-regulation of FTR1 enzyme in Fe-limited cells. Notably, we did not detect ferric reductase, an enzyme noted to reduce Fe^3+^ for uptake by the FET3-FTR transport system (see below).

### Polysaccharide biosynthesis and its potential role in Fe uptake

Multiple enzymes involved in polysaccharide biosynthesis were up-regulated in Fe-limited cultures. High concentrations of extracellular polysaccharides are produced by Fe-limited diatoms and have a role in increasing Fe bioavailability [71], [72]. Detailed examinations of controlled substrates in seawater demonstrated that the photoreduction of ferric iron (Fe^3+^) occurs in the presence of acidic sugars under UV light [73]–[75]. These findings led to the examination of photoreduction of Fe^3+^ in the presence of diatom polysaccharide exudates [76]. Steigenberger et al. [76] demonstrated that the combination of UV light, diatom exudates and reactive oxygen species reduced Fe^3+^ to ferrous iron (Fe^2+^) and the presence of the exudates further stabilized the Fe^2+^ for eventual biological uptake.

Typically, the redox state of iron in seawater is Fe^3+^, thus requiring reduction prior to biological import into the cell. This is frequently carried out by ferric reductase, which is part of the membrane-bound FET3-FTR transport system. Ferric reductase was not identified in our proteomic analysis because it is either in low abundance in the cells or it was not efficiently extracted from the cell because it is membrane-bound. The up-regulation of enzymes used to produce polysaccharides in the Fe-limited culture might have provided a stabilizing effect for Fe^2+^ in the surrounding seawater. Having more available ferrous iron for uptake could supplement a diminished or absent ferric reductase uptake pathway.

### Rapid sinking via aggregation

Although this study did not include the analysis of transparent exopolymer production during Fe-limited growth, several studies have reported a thickening or the extracellular matrix and mucus [58], [77]. Extracellular polysaccharides have also been observed to enhance the diatoms' ability to aggregate, or raft together [58], [77]–[79]. This rafting of cells is also observed in the open ocean when cells have reached stationary phase and is thought to be a contributor to increased organic C export to the ocean floor [80], [81]. Thus, the elevated production of polysaccharides by Fe-limited *T. pseudonana* could enhance the overall export rate of the cells out of the photic zone. In addition, this study, and others, have observed that when diatoms are Fe-limited they increase the thickness of the silica frustule and decrease the size of the cells (Table 2) [10], [58], [82], [83]. As a result, this increases the rate at which these diatom cells sink to the ocean floor. A rapid sinking rate from the upper ocean to the deep ocean might reduce bacterial colonization and the ultimate degradation rate of diatom organic matter. Consequently, those regions where diatoms are Fe-limited there should be enhanced algal C export from the upper ocean to deeper waters and sediments below. The identification of diatom photosystem proteins in sediment traps intercepting particles from the water column, and from ocean floor sediments, validates that their rapid vertical transport from the upper ocean does enhance C preservation [84], [85].

## Conclusion

Using comprehensive whole-cell proteomic approaches we evaluated metabolic acclimation of the diatom *T. pseudonana* to steady-state Fe limitation. When this diatom is acclimated to Fe limitation, the proteins expressed suggest that intracellular N and Fe recycling are used to conserve essential resources during mid-exponential growth. Up-regulation of transaminases and proteolytic enzymes allows the diatom cell to harvest N from amino acids without transporting equivalent quantities of N into the cells using N-specific transporters. Intracellular recycling would decrease the amount of C and N needed by the cell. The lack of Ni-ABC transporters, an enzyme found to be necessary for the function of the urease enzyme, suggests that the bioavailable urea is not being utilized when the diatom is Fe-limited. Here, we propose a strategy as to how Fe-limited diatoms might continue to grow with limited assimilation of new N.

When acclimated to low levels of Fe, this diatom is able to continue photosynthesis to generate ATP despite the high Fe requirements of photosynthetic electron transport. A decrease in PSII photosynthetic efficiency, as observed in this study (Table 2), is a signature characteristic of Fe-limitation [86]. Likewise, intracellular Fe concentrations are reduced when diatoms are Fe-limited [17], [54], [87]. This suggests that to maintain steady-state growth when Fe-limited, Fe must be conserved within the cell and unique mechanisms to acquire Fe may exist. We propose, based on previous experiments [76] and the proteins and pathways presented in this study, that increased extracellular polysaccharides combined with UV light and H_2_O_2_ production from heightened photorespiration might make ferrous Fe more bioavailable. That said, we also note that whole-cell proteomic profiling is not optimized for extracting membrane-bound proteins, thus possibly not providing some membrane-bound Fe-specific transporters.

Further adaptations for subsistence under Fe limitation include the enhanced use of the pentose phosphate pathway. This bypass of the glycolysis pathway yields substrates that are important to nucleotide, amino acid, and fatty acid synthesis. The intracellular storage and movement of N-containing compounds in these Fe-limited diatoms is one of the more unique and unanticipated results from this study. In addition, rather than the ammonia by-product resulting from photorespiration being a costly substrate to discard, diatoms might shuttle ammonia to the cytoplasm-located urea cycle enzymes. Although during Fe-limitation the enzymes necessary to complete the full urea cycle are not present, multiple enzymes suggest that the N-rich substrates are shunted from the cycle and packaged into arginine and low molecular weight polyamines. The up-regulation of spermine synthase suggests that polyamines, a key molecule required for silica precipitation [88], are in abundance in these Fe-limited diatoms. This would suggest that the thick silica frustule and enhanced sinking rate of Fe-limited diatoms might result from photorespiration by-products. These polyamines are also a reserve for N for subsequent utilization.

Here we have outlined seven essential cellular processes expressed in the proteome that are either enhanced or modified by diatoms acclimated to Fe-limited growth conditions. Using a detailed proteomic profiling method, it was possible to follow metabolic pathways that are modified and determine if nutrients or metabolic substrates were re-directed to maintain cellular homeostasis. Due to the rapid and dynamic nature of protein and transcript expression, acclimation to a defined nutrient condition is essential to avoid harvesting cells that have not fully acclimated physiologically to an environmental change. Given the proliferation of environmental manipulation experiments – for example in climate change research on oceanic biota [89] - it is also essential to report all details of growth conditions utilized.

## Supporting Information

Figure S1 **Metabolic biochemistry map and relative expression of proteins expressed and identified in Fe-limited** ***T. pseudonana*** **.** Maps include relative expression data from triplicate PAcIFIC analyses on a tandem mass spectrometer from *Thalassiosira pseudonana* acclimated to Fe-limitation. Each node (or corner) represents a metabolite and the lines connecting the nodes represent an enzyme. A colored line represents proteins that were identified in the particular cell state. The thickness of the line is a function of the number of unique peptides identified from that particular protein [line thickness = 5* log_2_(number of unique peptides identified)]. This function was applied to visually express the larger range of protein expression while maintaining a line width between 5–20 pixels. Metabolites were not measured in this study. Colors from top left – light blue: sugar and glycan biosynthesis, light purple: starch and sucrose metabolism (including photosynthesis, oxidative phosphorylation, carbon fixation), dark purple: glycolysis-gluconeogenesis (including TCA cycle), red: nucleotide metabolism, teal: lipid metabolism, orange: amino acid metabolism (including urea cycle).
(PNG)

Figure S2 **Metabolic biochemistry map and relative expression of proteins expressed and identified in Fe-replete** ***T. pseudonana*** **.** Maps include relative expression data from triplicate PAcIFIC analyses on a tandem mass spectrometer from *Thalassiosira pseudonana* acclimated to Fe-replete conditions. Each node (or corner) represents a metabolite and the lines connecting the nodes represent an enzyme. A colored line represents proteins that were identified in the particular cell state. The thickness of the line is a function of the number of unique peptides identified from that particular protein [line thickness = 5* log_2_(number of unique peptides identified)]. This function was applied to visually express the larger range of protein expression while maintaining a line width between 5–20 pixels. Metabolites were not measured in this study. Colors from top left – light blue: sugar and glycan biosynthesis, light purple: starch and sucrose metabolism (including photosynthesis, oxidative phosphorylation, carbon fixation), dark purple: glycolysis-gluconeogenesis (including TCA cycle), red: nucleotide metabolism, teal: lipid metabolism, orange: amino acid metabolism (including urea cycle).
(PNG)

Table S1 **Spectral counts, Qspec statistics, and functional annotations of proteins identified in all mass spectrometry experiments.**
(XLSX)

Table S2 **A list of proteins identified to be significantly up- or down- regulated in Fe-limited cells by** ***QSpec*** **from analyses of 4 biological splits from each culture.** Global ID number corresponds to NCBI identification of each protein (www.ncbi.nlm.nih.gov), Protein Function refers to the annotated and reported function of each protein sequence identified, QSpec Fold Change is calculated from the log_2_ of the spectral counts from Fe-limited cells divided by spectral counts from Fe-replete cells where the Bayes factor >10 criteria is met, and Cellular Processes refers to a generic term providing some, but not all, information regarding the function of the protein within the cell: TRANSLATION: RNA processing, protein building; DNA: DNA processing and binding; PHOTO: photosynthesis, light reactions; AAmet: amino acid metabolism; FA: fatty acid biosynthesis, CARBmet: carbohydrate metabolism; ENERGY: ATP metabolic process or glucose catabolic process; CMI: cell membrane integrity; REG: regulatory processes, signaling; IT: Intracellular trafficking of molecules; TRANSPORTER: transports molecules into cell; Glyc: glycolysis; PPP: pentose phosphate pathway; PROT_deg: protein degradation; CD: cell division; OXI-RED: oxidation-reduction.
(DOCX)

Table S3 **Spectral counts and Qspec statistics for proteins identified in all mass spectrometry experiments from the Lipopolysaccharide biosynthesis, Polysaccharide metabolism, spermine biosynthesis, silica precipitation pathways.**
(XLSX)

Table S4 **Spectral counts and Qspec statistics for proteins identified in all mass spectrometry experiments from the Pentose Phosphate pathway.**
(XLSX)

Table S5 **Spectral counts and Qspec statistics for proteins identified in all mass spectrometry experiments from the glycolysis or gluconeogenesis pathways.**
(XLSX)

Table S6 **Spectral counts and Qspec statistics for proteins identified in all mass spectrometry experiments from the Amino Acid Metabolism pathways.**
(XLSX)

Table S7 **Spectral counts and Qspec statistics for proteins identified in all mass spectrometry experiments from the Photosynthesis subunits and pathways.**
(XLSX)

Table S8 **Spectral counts and Qspec statistics for proteins identified in all mass spectrometry experiments from the Carbon Fixation pathways.**
(XLSX)

Text S1 **Directions and raw data for making iPath Figures online using enzyme commission numbers (EC numbers), or associated KEGG pathways.** This is a brief manual for submitting the raw data to generate more detailed and interactive maps identical to Figure S1 and S2 using iPath2.0. Raw data to input is at the end of this document. Input of data online will allow more detailed information on each enzyme to be viewed and examined.
(DOCX)
