# Supplementary figures and images for: Diatom Proteomics Reveals Unique Acclimation Strategies to Mitigate Fe Limitation
Source: PLoS One. 2013 Oct 16;8(10):e75653. doi: 10.1371/journal.pone.0075653 (PMC3797725; doi:10.1371/journal.pone.0075653)

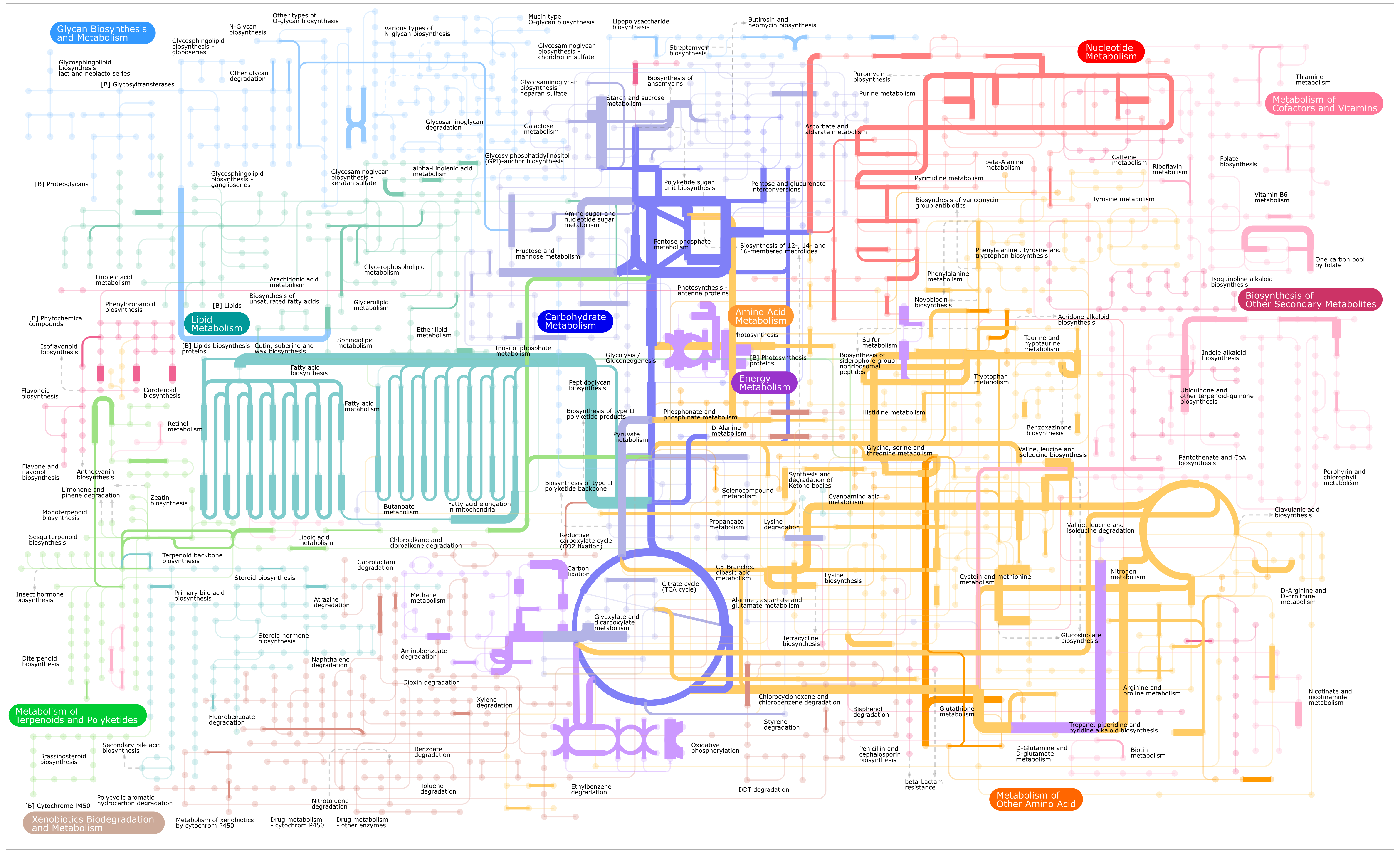

Supplement: Figure S1 — Metabolic biochemistry map and relative expression of proteins expressed and identified in Fe-limited T. pseudonana . Maps include relative expression data from triplicate PAcIFIC analyses on a tandem mass spectrometer from Thalassiosira pseudonana acclimated to Fe-limitation. Each node (or corner) represents a metabolite and the lines connecting the nodes represent an enzyme. A colored line represents proteins that were identified in the particular cell state. The thickness of the line is a function of the number of unique peptides identified from that particular protein [line thickness = 5* log2(number of unique peptides identified)]. This function was applied to visually express the larger range of protein expression while maintaining a line width between 5–20 pixels. Metabolites were not measured in this study. Colors from top left – light blue: sugar and glycan biosynthesis, light purple: starch and sucrose metabolism (including photosynthesis, oxidative phosphorylation, carbon fixation), dark purple: glycolysis-gluconeogenesis (including TCA cycle), red: nucleotide metabolism, teal: lipid metabolism, orange: amino acid metabolism (including urea cycle). (PNG) [file pone.0075653.s001.png]

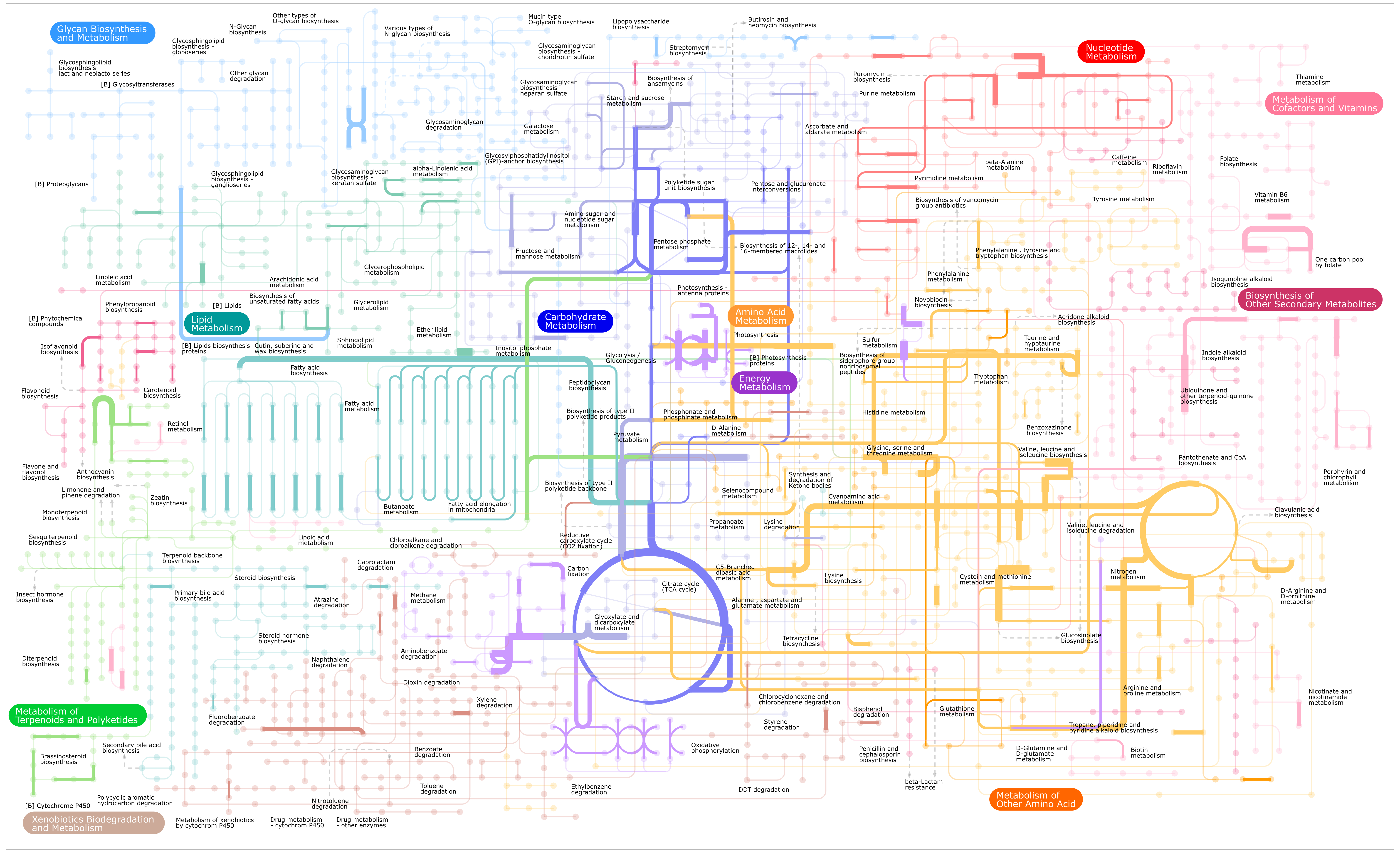

Supplement: Figure S2 — Metabolic biochemistry map and relative expression of proteins expressed and identified in Fe-replete T. pseudonana . Maps include relative expression data from triplicate PAcIFIC analyses on a tandem mass spectrometer from Thalassiosira pseudonana acclimated to Fe-replete conditions. Each node (or corner) represents a metabolite and the lines connecting the nodes represent an enzyme. A colored line represents proteins that were identified in the particular cell state. The thickness of the line is a function of the number of unique peptides identified from that particular protein [line thickness = 5* log2(number of unique peptides identified)]. This function was applied to visually express the larger range of protein expression while maintaining a line width between 5–20 pixels. Metabolites were not measured in this study. Colors from top left – light blue: sugar and glycan biosynthesis, light purple: starch and sucrose metabolism (including photosynthesis, oxidative phosphorylation, carbon fixation), dark purple: glycolysis-gluconeogenesis (including TCA cycle), red: nucleotide metabolism, teal: lipid metabolism, orange: amino acid metabolism (including urea cycle). (PNG) [file pone.0075653.s002.png]
