# Supplementary material for: Diatom Proteomics Reveals Unique Acclimation Strategies to Mitigate Fe Limitation
Source: PLoS One. 2013 Oct 16;8(10):e75653. doi: 10.1371/journal.pone.0075653 (PMC3797725; doi:10.1371/journal.pone.0075653)
Supplement: Table S2 — A list of proteins identified to be significantly up- or down- regulated in Fe-limited cells by QSpec from analyses of 4 biological splits from each culture. Global ID number corresponds to NCBI identification of each protein (www.ncbi.nlm.nih.gov), Protein Function refers to the annotated and reported function of each protein sequence identified, QSpec Fold Change is calculated from the log2 of the spectral counts from Fe-limited cells divided by spectral counts from Fe-replete cells where the Bayes factor >10 criteria is met, and Cellular Processes refers to a generic term providing some, but not all, information regarding the function of the protein within the cell: TRANSLATION: RNA processing, protein building; DNA: DNA processing and binding; PHOTO: photosynthesis, light reactions; AAmet: amino acid metabolism; FA: fatty acid biosynthesis, CARBmet: carbohydrate metabolism; ENERGY: ATP metabolic process or glucose catabolic process; CMI: cell membrane integrity; REG: regulatory processes, signaling; IT: Intracellular trafficking of molecules; TRANSPORTER: transports molecules into cell; Glyc: glycolysis; PPP: pentose phosphate pathway; PROT_deg: protein degradation; CD: cell division; OXI-RED: oxidation-reduction. (DOCX) [file pone.0075653.s004.docx]

**Table S2. A list of proteins identified to be significantly up- or down- regulated in Fe-limited cells by *QSpec* from analyses of 4 biological splits from each culture.**

|  |  | **Global ID number** | **Protein Function** | ***QSpec*** | **Cellular Processes** |
| --- | --- | --- | --- | --- | --- |
|  |  |  |  | **Protein abundance**  **Log_2_ fold change** |  |
|  | **Down-regulated proteins in Fe-limited cells** | 224015655 | nickel ABC transporter periplasmic protein | -2.87 | **TRANSPORTER,AAmet** |
|  |  | 118411185 | 30S ribosomal protein S4 | -1.63 | **TRANSLATION** |
|  |  | 223994385 | chloroplast 50S ribosomal protein L15 | -1.61 | **TRANSLATION** |
|  |  | 223993577 | Predicted Protein | -1.38 |  |
|  |  | 209583663 | Predicted protein B0432.4 | -1.26 |  |
|  |  | 223998678 | Phosphoenolpyruvate carboxylase (PEPC2) | -1.24 | **PHOTO, ENERGY** |
|  |  | 118411124 | 50S ribosomal protein L1 | -1.21 | **TRANSLATION** |
|  |  | 224007537 | ATP binding | -1.13 | **DNA, REG, ENERGY** |
|  |  | 224002675 | ribosomal protein L22 | -1.12 | **TRANSLATION** |
|  |  | 224010633 | COG1233: Phytoene dehydrogenase and related proteins | -1.09 | **OXI-RED** |
|  |  | 224009938 | RsuA 16S rRNA U516 pseudouridine synthase | -1.08 | **TRANSLATION** |
|  |  | 118411203 | 50S ribosomal protein L5 | -1.08 | **TRANSLATION** |
|  |  | 223994299 | Predicted proteins -PFAM: HEC/Ndc80p family | -0.98 | **DNA** |
|  |  | 118411141 | cell division protein FtsH-like protein | -0.96 | **CD, DNA PROT_deg** |
|  |  | 223993621 | proteophosphoglycan ppg4 | -0.95 | **CMI, CARBmet** |
|  |  | 223995685 | Predicted cell division protein FtsH2 | -0.88 | **CD, DNA PROT_deg** |
|  |  | 118411191 | 50S ribosomal protein L4 | -0.85 | **TRANSLATION** |
|  |  | 118411193 | 50S ribosomal protein L2 | -0.84 | **TRANSLATION** |
|  |  | 209583588 | SHM1 glycine hydroxymethyltransferase | -0.82 | **AAmet** |
|  |  | 224007497 | Predicted: nicotinamide nucleotide transhydrogenase | -0.82 | **OXI-RED** |
|  |  | 118411222 | 30S ribosomal protein S6 | -0.82 | **TRANSLATION** |
|  |  | 209586173 | Ribosomal protein L3 | -0.81 | **TRANSLATION** |
|  |  | 223993421 | Muc19: mucin 19 | -0.81 | **CMI** |
|  |  | 223996073 | phosphoribosylpyrophosphate synthetase | -0.8 | **PPP** |
|  |  | 118411201 | 50S ribosomal protein L14 | -0.8 | **TRANSLATION** |
|  |  | 223998400 | heat shock protein 83 | -0.79 | **REG** |
|  |  | 118411123 | 50S ribosomal protein L11 | -0.78 | **TRANSLATION** |
|  |  | 224008965 | 60S ribosomal protein L6 CgRPL6 | -0.78 | **TRANSLATION** |
|  |  | 224006742 | 30S ribosomal protein S1 | -0.77 | **TRANSLATION** |
|  |  | 118411126 | 30S ribosomal protein S2 | -0.77 | **TRANSLATION** |
|  |  | 224014104 | Predicted protein | -0.76 |  |
|  |  | 224015517 | Predicted protein- DNA binding domain | -0.73 | **DNA** |
|  |  | 224013552 | pyruvate dehydrogenase (lipoamide) beta | -0.72 | **Glyc, AAmet** |
|  |  | 223999739 | chloroplast light harvesting protein isoform 12 | -0.7 | **PHOTO** |
|  |  | 224009568 | Predicted protein | -0.67 |  |
|  |  | 224009197 | Predicted protein similar to CG9888-PA | -0.67 | **TRANSLATION** |
|  |  | 118411109 | ATP synthase CF0 B' chain subunit II | -0.64 | **ENERGY** |
|  |  | 224015308 | Photosystem I light harvesting proteins | -0.64 | **PHOTO** |
|  |  | 223995405 | Predicted protein | -0.64 |  |
|  |  | 118411155 | cytochrome b6-f complex subunit IV | -0.63 | **OXI-RED, PHOTO** |
|  |  | 223998931 | adenosinetriphosphatase | -0.63 | **ENERGY** |
|  |  | 118411180 | photosystem II reaction center protein D1 | -0.61 | **PHOTO** |
|  |  | 224001616 | ATPase, E1-E2 type | -0.58 | **ENERGY** |
|  |  | 118411153 | photosystem I ferredoxin-binding protein | -0.58 | **PHOTO** |
|  |  | 118411220 | ATP-dependent clp protease ATP-binding subunit | -0.58 | **ENERGY** |
|  |  | 223996813 | ribosomal protein S4, Y-linked | -0.57 | **TRANSLATION** |
|  |  | 223992923 | Predicted Porin protein | -0.57 | **TRANSPORTER** |
|  |  | 118411137 | cytochrome f | -0.56 | **PHOTO** |
|  |  | 118411190 | 50S ribosomal protein L3 | -0.56 | **TRANSLATION** |
|  |  | 224003409 | Predicted Protein No BLAST result | -0.54 |  |
|  |  | 118411135 | ATP synthase CF1 epsilon chain | -0.54 | **ENERGY** |
|  |  | 224010635 | geranyl-geranyl reductase | -0.53 | **OXI-RED** |
|  |  | 224003107 | oxygen-evolving enhancer protein 1 precursor | -0.51 | **PHOTO** |
|  |  | 118411112 | ATP synthase CF1 alpha chain | -0.5 | **ENERGY** |
|  | **Up-regulated proteins in Fe-limited cells** | 224005154 | phosphoglycerate kinase precursor | 0.5 | **Glyc** |
|  |  | 224011888 | predicted translation elongation factor G | 0.5 | **TRANSLATION, CD** |
|  |  | 223994191 | Predicted protein | 0.59 |  |
|  |  | 224002408 | aconitate hydratase 2 (citrate hydro-lyase 2) (aconitase 2) | 0.59 | **CARBmet, ENERGY** |
|  |  | 223993043 | glyceraldehyde-3-phosphate dehydrogenase precursor | 0.6 | **Glyc** |
|  |  | 224009658 | phosphoadenosine-phosphosulphate reductase | 0.6 | **OXI-RED, ENERGY** |
|  |  | 223993867 | putative CDC48/ATPase | 0.63 | **ENERGY** |
|  |  | 224000661 | fructose-1,6-bisphosphate aldolase precursor | 0.65 | **Glyc** |
|  |  | 223996511 | Spermine synthase | 0.66 | **CMI** |
|  |  | 209583455 | copper-induced girdle band-associated cell surface protein | 0.67 | **CMI** |
|  |  | 223998614 | Predicted protein | 0.68 |  |
|  |  | 223999927 | Phosphoglucomutase, cytoplasmic (PGM) | 0.69 | **Glyc** |
|  |  | 223997268 | manganese superoxide dismutase | 0.69 | **OXI-RED** |
|  |  | 223999217 | phosphoribulokinase | 0.71 | **ENERGY** |
|  |  | 224012331 | ascorbate peroxidase | 0.73 | **CARBmet, AAmet** |
|  |  | 223993279 | 2-isopropylmalate synthase A | 0.73 | **AAmet** |
|  |  | 118411218 | translation elongation factor Tu | 0.76 | **TRANSLATION, CD** |
|  |  | 223999031 | CbbX protein homolog | 0.76 | **ENERGY** |
|  |  | 224007002 | Adenosine kinase | 0.77 | **TRANSLATION** |
|  |  | 224007705 | elongation factor alpha-like protein | 0.77 | **TRANSLATION, CD** |
|  |  | 223993031 | Fe transporter Ftr1 | 0.78 | **TRANSPORTER, IT** |
|  |  | 224010100 | histone H2A.1 | 0.79 | **REG** |
|  |  | 224001660 | GDP-mannose dehydratase | 0.82 | **CARBmet** |
|  |  | 224013212 | fucoxanthin chlorophyll a/c binding protein | 0.87 | **PHOTO** |
|  |  | 224012529 | HSP70-like protein | 0.87 | **CMI** |
|  |  | 224000862 | putative signal-transduction protein with CBS domains | 0.87 | **REG** |
|  |  | 223992617 | putative aminotransferase AGD2 | 0.87 | **AAmet** |
|  |  | 223995719 | membrane-associated 30 kD protein-like | 0.89 | **CMI, REG** |
|  |  | 223999607 | clathrin binding | 0.91 | **IT** |
|  |  | 224013082 | N-acetylornithine aminotransferase | 0.91 | **AAmet** |
|  |  | 224013261 | Predicted 3-oxoacyl-[acyl-carrier-protein] synthase | 0.92 | **FA** |
|  |  | 118411104 | ribulose-1,5-bisphosphate carboxylase/oxygenase large subunit | 0.94 | **PHOTO** |
|  |  | 224001930 | COG0473: Isocitrate/isopropylmalate dehydrogenase | 1.02 | **AAmet** |
|  |  | 224002995 | elongation factor 2 | 1.03 | **TRANSLATION** |
|  |  | 224001430 | ATPREP1/ATZNMP; metalloendopeptidase | 1.07 | **PROT_deg** |
|  |  | 224006554 | Tubulin alpha-2 chain | 1.1 | **CMI, IT** |
|  |  | 209583661 | GTPAse: T06D8.1b | 1.1 | **REG, CD, IT** |
|  |  | 224010070 | serine hydroxymethyltransferase | 1.14 | **AAmet** |
|  |  | 223993519 | ADP-ribosylation factor | 1.14 | **IT, REG** |
|  |  | 224013196 | stress-inducible protein STI1 homolog | 1.17 | **REG** |
|  |  | 223999013 | protein kinase, putative | 1.18 | **CARBmet, AAmet** |
|  |  | 224001286 | 2nd Hit Predicted protein CBG01077 | 1.19 | **DNA** |
|  |  | 118411164 | Rubisco expression protein | 1.19 | **PHOTO** |
|  |  | 223992817 | predicted protein | 1.21 |  |
|  |  | 224003531 | Pyruvate dehydrogenase | 1.21 | **AAmet, Glyc, OXI-RED** |
|  |  | 223999929 | UDP-glucose pyrophosphorylase | 1.23 | **CARBmet, PPP** |
|  |  | 209583468 | copper-induced girdle band-associated cell surface protein | 1.24 | **CMI, CD** |
|  |  | 223998024 | 14-3-3 regulatory protein | 1.25 | **REG** |
|  |  | 223994125 | cysD: ATP-sulfurylase | 1.3 | **ENERGY** |
|  |  | 223993693 | Predicted: Bromodomain; RING3 | 1.35 | **CD, TRANSLATION** |
|  |  | 223997516 | Aspartate-semialdehyde dehydrogenase, USG-1 related | 1.36 | **AAmet** |
|  |  | 224001278 | enolase | 1.37 | **Glyc** |
|  |  | 223994119 | Predicted HSP70 heat shock 70kD protein 4 | 1.39 | **REG** |
|  |  | 224001636 | predicted protein cgd4_200 | 1.39 |  |
|  |  | 223996962 | proliferating cell nuclear antigen | 1.49 | **DNA** |
|  |  | 223999219 | Phosphofructokinase | 1.5 | **Glyc** |
|  |  | 224008957 | Predicted protein: ProSite- Protamine P1 signature | 1.53 | **CD, AAmet** |
|  |  | 224008733 | similar to coatomer protein complex, subunit gamma 2 | 1.54 | **TRANS** |
|  |  | 223993357 | Tubulin beta chain (Beta tubulin) | 1.56 | **CMI** |
|  |  | 223995669 | Predicted RNA Helicase | 1.58 | **TRANSLATION** |
|  |  | 224013856 | Hmg protein 1.2, isoform c | 1.6 | **DNA** |
|  |  | 223999447 | asparagine synthetase | 1.63 | **AAmet** |
|  |  | 224009908 | aspartate-ammonia ligase | 1.63 | **AAmet** |
|  |  | 223997294 | fructose-bisphosphate aldolase | 1.65 | **Glyc** |
|  |  | 209586218 | Predicted protein related to NonF protein | 1.68 | **REG, PROT_deg** |
|  |  | 224012695 | ClpB protein | 1.7 | **PROT_deg** |
|  |  | 224014576 | COG0166: Glucose-6-phosphate isomerase | 1.75 | **Glyc** |
|  |  | 223997294 | fructose-1,6-biphosphate aldolase precursor | 1.78 | **Glyc** |
|  |  | 224015421 | phosphoesterase | 1.79 | **AAmet** |
|  |  | 224009534 | UDP-glucose 6-dehydrogenase | 1.8 | **OXI-RED, PPP** |
|  |  | 224010838 | metalloendopeptidase | 1.81 | **PROT_deg** |
|  |  | 223996381 | chloroplast cysteine synthase 1 precursor | 1.87 | **AAmet** |
|  |  | 224013436 | Predicted protein MG01320.4 | 1.89 | **CARBmet** |
|  |  | 223993045 | transketolase | 1.91 | **PPP** |
|  |  | 224013544 | Predicted protein | 1.94 |  |
|  |  | 224012106 | Predicted protein UM04584.1 | 2.14 | **AAmet** |
|  |  | 223997748 | Predicted protein | 2.3 | **OXI-RED** |
