# Supplementary material for: Diatom Proteomics Reveals Unique Acclimation Strategies to Mitigate Fe Limitation
Source: PLoS One. 2013 Oct 16;8(10):e75653. doi: 10.1371/journal.pone.0075653 (PMC3797725; doi:10.1371/journal.pone.0075653)
Supplement: Text S1 — Directions and raw data for making iPath Figures online using enzyme commission numbers (EC numbers), or associated KEGG pathways. This is a brief manual for submitting the raw data to generate more detailed and interactive maps identical to Figure S1 and S2 using iPath2.0. Raw data to input is at the end of this document. Input of data online will allow more detailed information on each enzyme to be viewed and examined. (DOCX) [file pone.0075653.s011.docx]

**Directions for making iPath Figures using proteomic data from Nunn et al. 2013**

This is a brief manual for submitting the raw data to generate more detailed and interactive maps identical to Figure S1 and S2 using iPath2.0. Raw data to input is at the end of this document.

Go to <http://pathways.embl.de/>
Click on the image to select **iPath v 2: the main interface.**


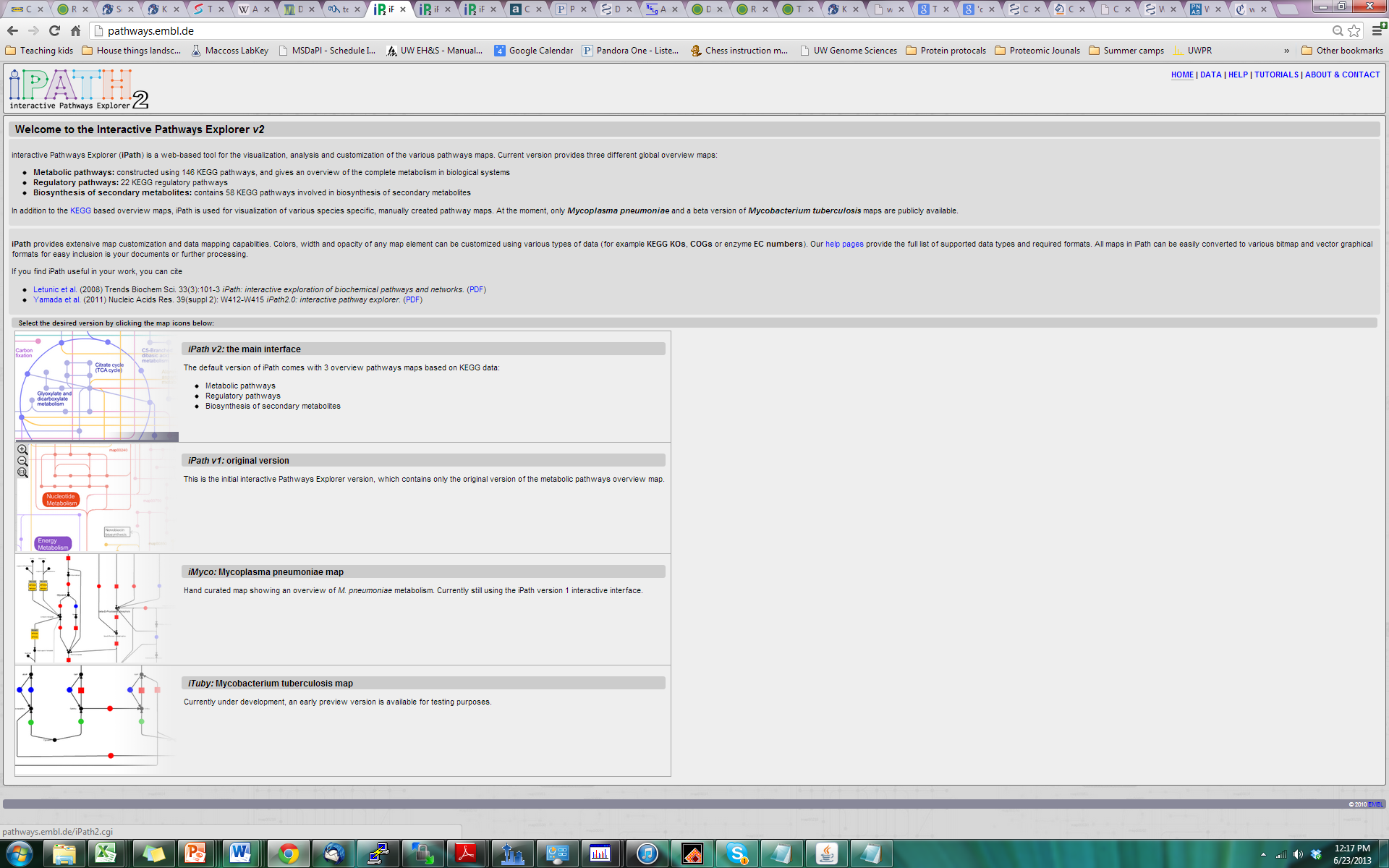

On the upper right corner select "Customize"


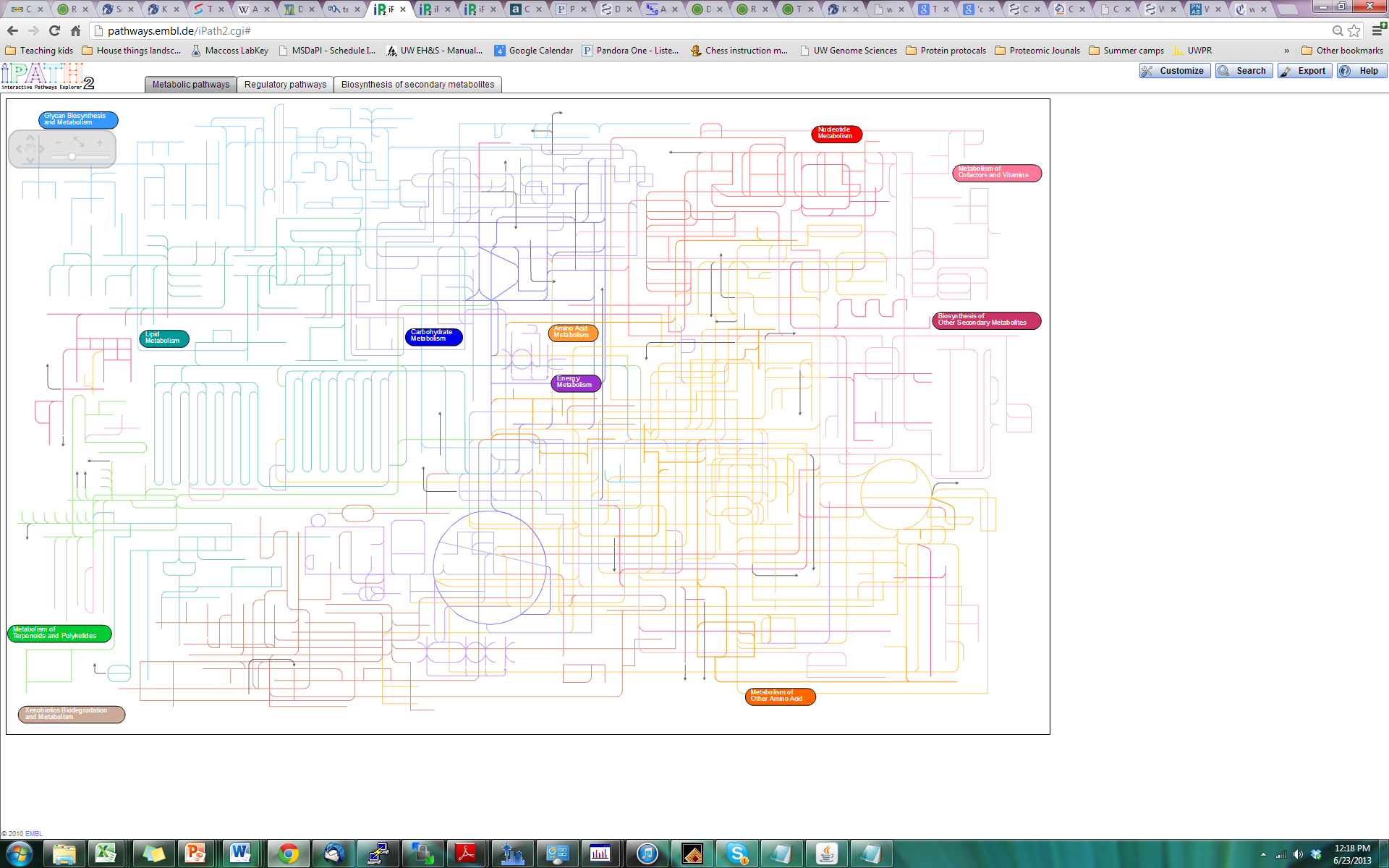


This is where you can upload data to generate an iPath map
You can provide a title to the plot by typing in the “Selection title” space


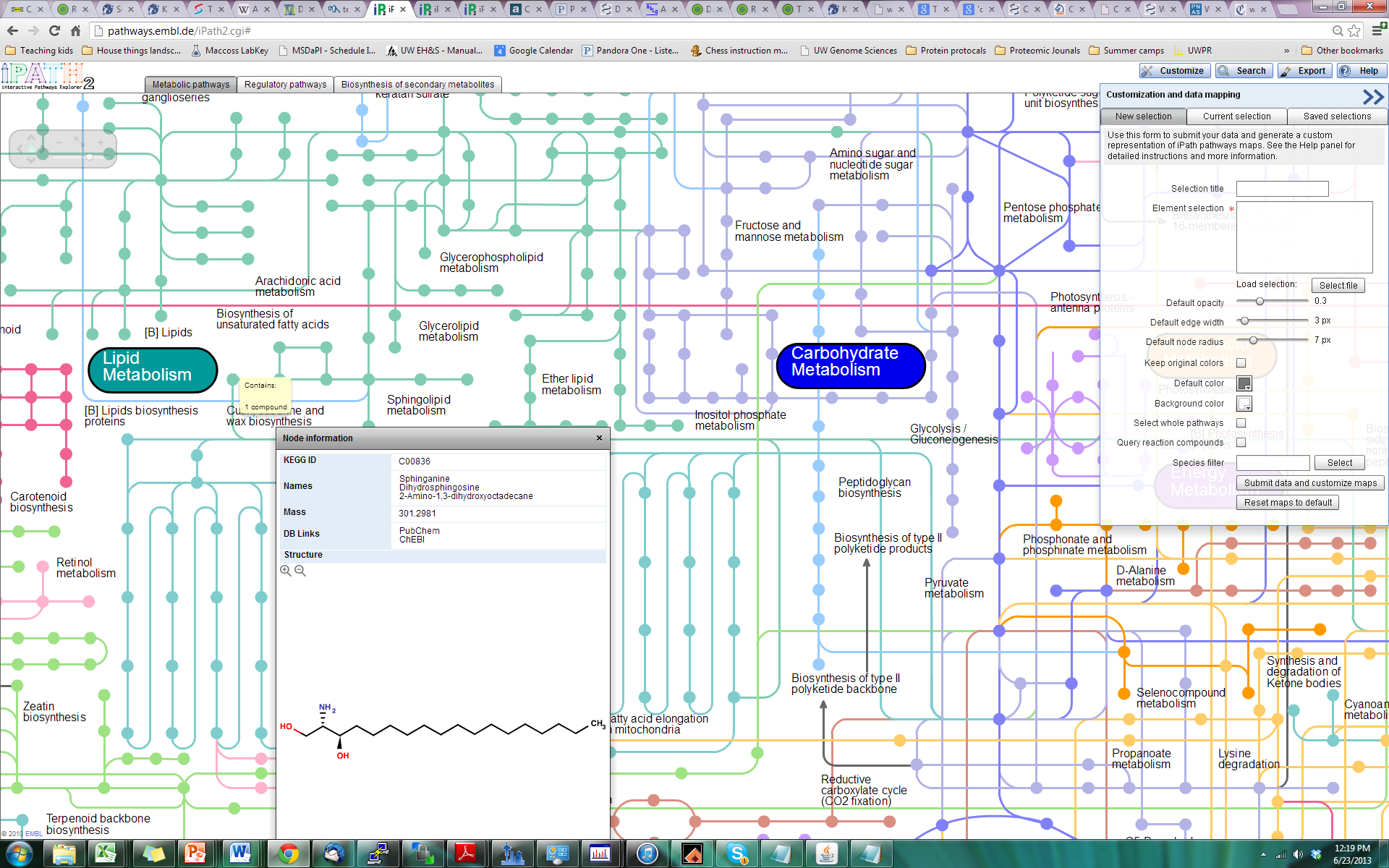

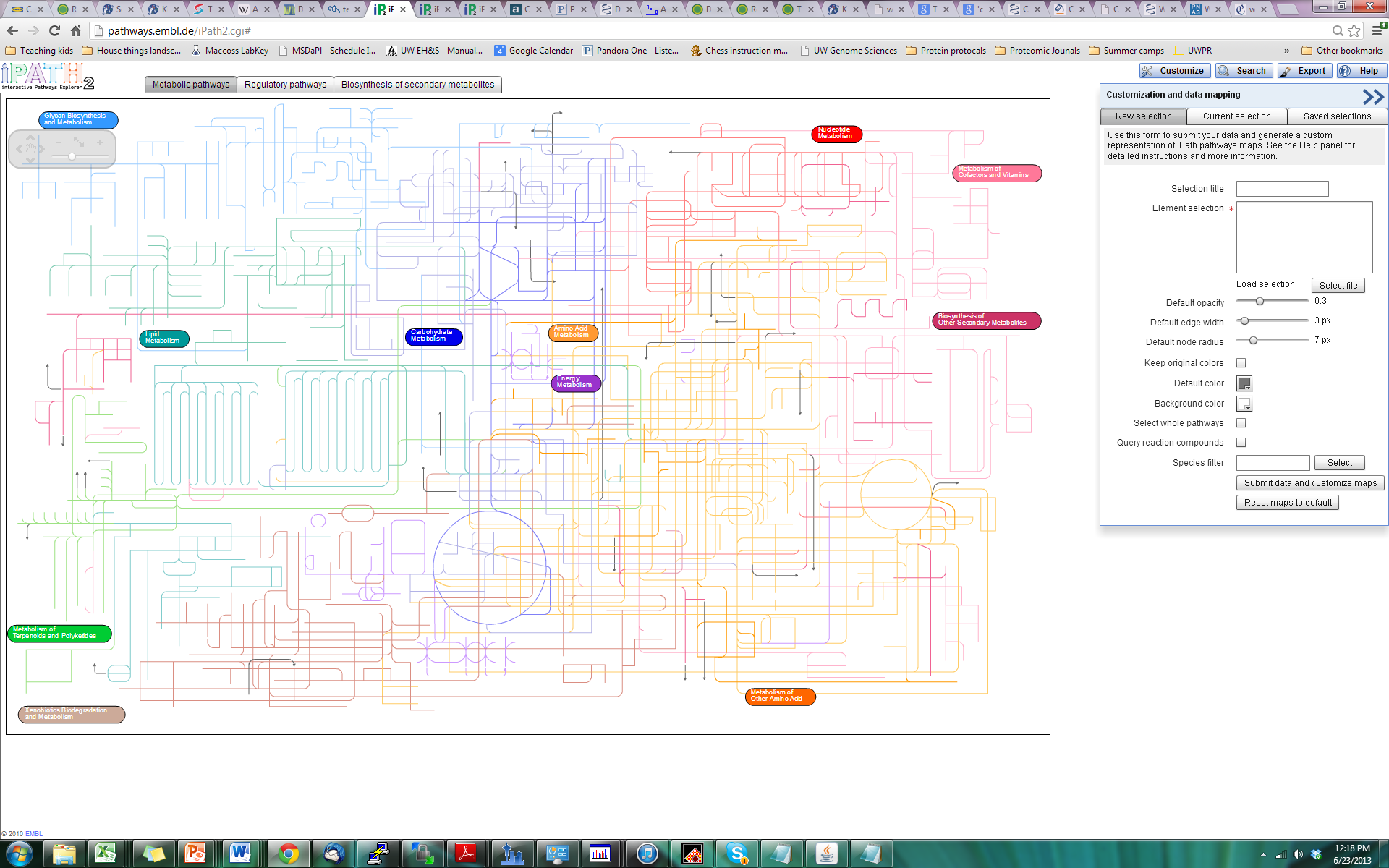

Copy the data that is listed below (at the end of this document) under either Fe-limited or Fe-replete and paste it into the window “Element Selection:. Do not change formatting. You can also save the lists at the bottom of the page as .txt files and upload them directly from your hard-drive using the “Select File” button.

Check the box labeled "keep original colors"
Click button for "Submit data and customize map"
Wait about 20 seconds for the map to be generated.

Now you can zoom in on the proteins identified that are present in the metabolic biochemistry map.  Each line represents an enzyme and the nodes represent compounds. The upper left-hand corner has a picture of a "+" and "-" sign to zoom in and out or you can select the hand to push the sheet of paper around to look at different areas while you are zoomed in.
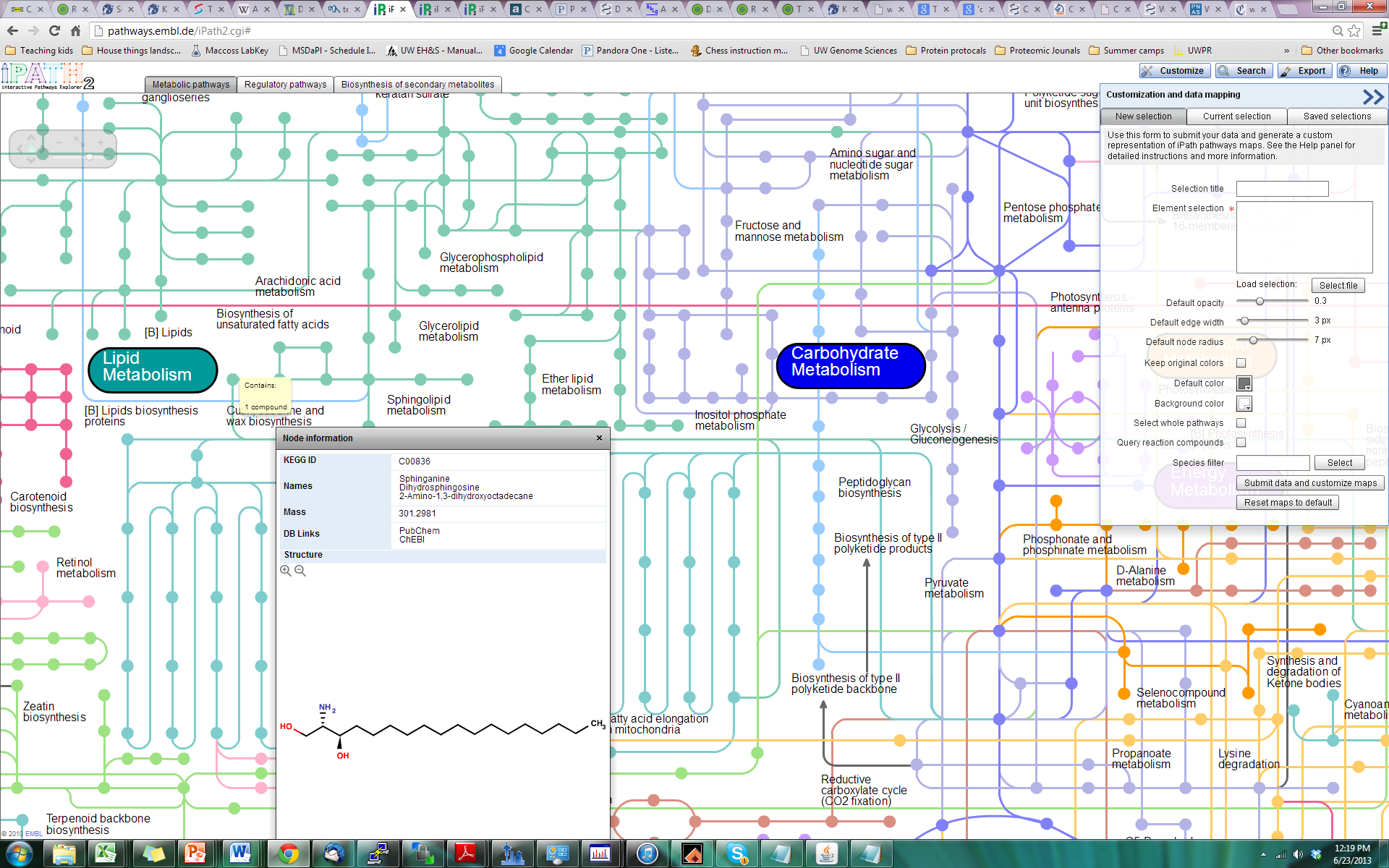

If you take your mouse pointer and hover over a line, you will see that a list of enzymes will be highlighted.  You can then select a specific identified enzyme to get more information on that protein. You can also select nodes (dots on the plot).  These dots represent different compounds that are synthesized by the enzyme that is represented by the line.

Fe-limited data- copy list below (end at the *) do not change formatting

K02637 1 W5

E1 1 W5

K01419 1 W5

E5.3.4.1 1 W5

K01103 1 W5

K07376 1 W5

K01829 1 W5

K00794 1 W5

E3.2.1.18 1 W5

K03667 1 W5

E3.2.1.18 1 W5

E1 1 W5

K04083 1 W5

E6.3.2.4 1 W5

E1 1 W5

K01426 1 W5

K02838 1 W5

E6.3.2.- 1 W5

E3.2.1.18 1 W5

K07200 1 W5

E5.3.4.1 1 W5

K04487 1 W5

E3 1 W5

E2.7.11.18 1 W5

E1 1 W5

K06980 1 W5

E3.2.1.18 1 W5

K08056 1 W5

K00927 1 W5

K00799 1 W5

K03076 1 W5

K04518 1 W5

K01893 1 W5

K01897 1 W5

K01549 1 W5

K00226 1 W5

E1.1.1.- 1 W5

K00391 1 W5

K03189 1 W5

K06972 1 W5

K02938 1 W5

K01086 1 W5

E3.6.3.14 1 W5

K08493 1 W5

K01530 1 W5

K03106 1 W5

K07517 1 W5

K00801 1 W5

E1.8.1.4 1 W5

E3.6.5.3 1 W5

E2.4.2.11 1 W5

K09838 1 W5

K01875 1 W5

K01463 1 W5

K06269 1 W5

K00698 1 W5

K08869 1 W5

K03694 1 W5

K08517 1 W5

E1 1 W5

E1.10.2.2 1 W5

E1.10.2.2 1 W5

E1.8.1.9 1 W5

K03977 1 W5

K01149 1 W5

E2.1.1 1 W5

K02738 1 W5

K00326 1 W5

K01267 1 W5

K09554 1 W5

K00863 1 W5

K03111 1 W5

K00762 1 W5

K09560 1 W5

K01770 1 W5

K01299 1 W5

K00301 1 W5

E1 1 W5

E5.2.1.8 1 W5

E1.11.1.5 1 W5

K06185 1 W5

K00384 1 W5

K00789 1 W5

K03263 1 W5

K09503 1 W5

K00765 1 W5

K05665 1 W5

K01802 1 W5

K00383 1 W5

E2.3.1.- 1 W5

K05765 1 W5

E1.3.1.33 1 W5

K03754 1 W5

K01876 1 W5

K00540 1 W5

E3.2.1.18 1 W5

K01358 1 W5

E3.2.1.18 1 W5

K01758 1 W5

K01423 1 W5

K03241 1 W5

K02959 1 W5

K09578 1 W5

K02921 1 W5

K04796 1 W5

K02924 1 W5

E1.1.1 1 W5

K07976 1 W5

K03177 1 W5

K03439 1 W5

E3.6.5.3 1 W5

E5.2.1.8 1 W5

K03868 1 W5

E5.3.99.2 1 W5

K08486 1 W5

K00616 1 W5

K03671 1 W5

K01265 1 W5

K03150 1 W5

K01809 1 W5

K01187 1 W5

E1.14.11.2 1 W5

E3.4.24 1 W5

K03097 1 W5

K00263 1 W5

E1.3.1.13 1 W5

K01784 1 W5

E2.7.1.- 1 W5

K00721 1 W5

E5.2.1.8 1 W5

E2.5.1.3 1 W5

K00899 1 W5

K00357 1 W5

E1.3.1.28 1 W5

K00212 1 W5

E1.1.1.65 1 W5

E5.2.1.8 1 W5

E6.3.2.4 1 W5

K07748 1 W5

K03131 1 W5

E1.4.99.1 1 W5

E3.4.19.1 1 W5

K03259 1 W5

K00850 1 W5

E3.6.1.3 1 W5

E3.6.1.3 1 W5

E2.1.1.127 1 W5

E5.2.1.8 1 W5

K01920 1 W5

E3.1.1.47 1 W5

K06119 1 W5

K01874 1 W5

K00837 1 W5

K02291 1 W5

K02013 1 W5

E2.7.1.37 1 W5

K00244 1 W5

E2 1 W5

K06067 1 W5

E5.3.1.9 1 W5

E2.7.1.37 1 W5

E3.2.1.18 1 W5

E3.2.1.18 1 W5

K00359 1 W5

K01028 1 W5

K01265 1 W5

E3.4.23.- 1 W5

K01284 1 W5

K00599 1 W5

K01303 1 W5

K01533 1 W5

K00697 1 W5

E2.3.1.74 1 W5

K01511 1 W5

E2.1.2.10 1 W5

K01850 1 W5

E2.3.1.48 1 W5

K03798 1 W5

E3.2.1.18 1 W5

K01872 1 W5

E3.1.1.3 1 W5

E3.2.1.3 1 W5

K03876 1 W5

K03107 1 W5

E2.7.1.37 1 W5

E5.2.1.8 1 W5

E2.3.1.48 1 W5

E6.3.2.- 1 W5

E3.6.1.3 1 W5

K01516 1 W5

E2.7.1.37 1 W5

K03456 1 W5

E3.2.1.3 1 W5

K00936 1 W5

K01878 1 W5

K02999 1 W5

E3.2.1.18 1 W5

K03240 1 W5

E3.2.1.3 1 W5

E6.3.2.19 1 W5

K03046 1 W7.92

K02694 1 W7.92

K02694 1 W7.92

K02723 1 W7.92

K02723 1 W7.92

K02891 1 W7.92

K02891 1 W7.92

K02997 1 W7.92

K02256 1 W7.92

K02261 1 W7.92

K03237 1 W7.92

E2.7.11.1 1 W7.92

K00812 1 W7.92

K00940 1 W7.92

K00697 1 W7.92

K06889 1 W7.92

K03006 1 W7.92

E3.4.21.92 1 W7.92

K02356 1 W7.92

K03839 1 W7.92

K03671 1 W7.92

K02976 1 W7.92

K00329 1 W7.92

K02971 1 W7.92

E6.3.2.19 1 W7.92

K03937 1 W7.92

E3.6.5.3 1 W7.92

K01165 1 W7.92

K01072 1 W7.92

K03940 1 W7.92

K01109 1 W7.92

E3.1.1.47 1 W7.92

K08727 1 W7.92

E2.3.1.48 1 W7.92

E3.1.3.41 1 W7.92

E1.3.1.33 1 W7.92

K01931 1 W7.92

K03031 1 W7.92

K00845 1 W7.92

K03715 1 W7.92

K07466 1 W7.92

K00891 1 W7.92

E2.5.1.16 1 W7.92

K01870 1 W7.92

K02885 1 W7.92

K04563 1 W7.92

E1.3.1.- 1 W7.92

K00795 1 W7.92

K03250 1 W7.92

K03243 1 W7.92

K05679 1 W7.92

E2.7.11.22 1 W7.92

E3.6.5.3 1 W7.92

K03076 1 W7.92

K03264 1 W7.92

K02978 1 W7.92

K01803 1 W7.92

K07575 1 W7.92

K03564 1 W7.92

K02876 1 W7.92

K02503 1 W7.92

K02958 1 W7.92

E2.7.1.- 1 W7.92

K03841 1 W7.92

E1.8.1.9 1 W7.92

K03100 1 W7.92

K03955 1 W7.92

K00036 1 W7.92

K03257 1 W7.92

K01637 1 W7.92

K01784 1 W7.92

K00559 1 W7.92

K02426 1 W7.92

K00658 1 W7.92

K03029 1 W7.92

E2.1.1.95 1 W7.92

K03033 1 W7.92

K02735 1 W7.92

K03094 1 W7.92

K02979 1 W7.92

K01736 1 W7.92

K09391 1 W7.92

K07127 1 W7.92

K01529 1 W7.92

K01090 1 W7.92

K00826 1 W7.92

K01529 1 W7.92

K03596 1 W7.92

E6.1.1.3 1 W7.92

K04382 1 W7.92

K03038 1 W7.92

E3.2.1.3 1 W7.92

K01754 1 W7.92

K01885 1 W7.92

E6.3.2.- 1 W7.92

K01735 1 W7.92

E3.6.1.- 1 W7.92

E3.6.1.3 1 W7.92

K00592 1 W7.92

E2.7.4.6 1 W7.92

E3.6.5.3 1 W7.92

K03941 1 W7.92

K01362 1 W7.92

K05805 1 W7.92

K01325 1 W7.92

K09550 1 W7.92

K08903 1 W7.92

K01520 1 W7.92

E2.3.1.12 1 W7.92

K08506 1 W7.92

E4.1.1.48 1 W7.92

K07566 1 W7.92

K06944 1 W7.92

E3.1.3.7 1 W7.92

E1 1 W7.92

E2.7.7.50 1 W7.92

K02357 1 W7.92

K03239 1 W7.92

K05768 1 W7.92

K01496 1 W7.92

K01825 1 W7.92

E1 1 W7.92

E2.7.7.6 1 W7.92

K03627 1 W7.92

K06158 1 W7.92

K01881 1 W7.92

K01887 1 W7.92

K08869 1 W7.92

E2.3.1.48 1 W7.92

K00624 1 W7.92

E3.6.5.1 1 W7.92

K10356 1 W7.92

K06948 1 W7.92

E1 1 W7.92

E3.4.24 1 W7.92

K07508 1 W7.92

E2.1.1.- 1 W7.92

E3.6.1.- 1 W7.92

K01099 1 W7.92

K01354 1 W7.92

K06957 1 W7.92

K01848 1 W7.92

E3.2.1.18 1 W7.92

K02709 1 W10

K02709 1 W10

K03043 1 W10

K03043 1 W10

K02949 1 W10

K02040 1 W10

K02125 1 W10

K03936 1 W10

K03935 1 W10

E2.7.1.- 1 W10

E1.5.1.2 1 W10

K02730 1 W10

K06027 1 W10

K01262 1 W10

E3.6.1.3 1 W10

E4.1.1.29 1 W10

K01883 1 W10

K01358 1 W10

K03039 1 W10

K02903 1 W10

K02956 1 W10

K02943 1 W10

E3.2.1.18 1 W10

E2.3.1.12 1 W10

K01829 1 W10

E3.2.1.3 1 W10

K03265 1 W10

E1 1 W10

E1 1 W10

K10251 1 W10

K03255 1 W10

E2.7.11.22 1 W10

E6.3.2.19 1 W10

K09496 1 W10

K00820 1 W10

E3.1.-.- 1 W10

K09498 1 W10

E5.4.2.8 1 W10

K09838 1 W10

E1.10.2.2 1 W10

K09272 1 W10

K00928 1 W10

K07515 1 W10

K05850 1 W10

K03100 1 W10

K06063 1 W10

K09015 1 W10

K02997 1 W10

K08678 1 W10

K03531 1 W10

K00164 1 W10

E1.14.11 1 W10

K01834 1 W10

K00872 1 W10

K00831 1 W10

K02500 1 W10

K02983 1 W10

K00813 1 W10

E5.1.3.3 1 W10

K00939 1 W10

K02838 1 W10

K00133 1 W10

K01267 1 W10

K01783 1 W10

K01783 1 W10

K01783 1 W10

K00344 1 W10

K00800 1 W10

K03778 1 W10

K09494 1 W10

K03030 1 W10

K01802 1 W10

K00324 1 W10

K03037 1 W10

E3.4.11.18 1 W10

K03266 1 W10

E5.2.1.8 1 W10

K08675 1 W10

K01802 1 W10

E1.11.1.5 1 W10

K00134 1 W10

K08906 1 W10

K03238 1 W10

K02951 1 W10

K03455 1 W10

K03704 1 W10

K03010 1 W10

K06067 1 W10

K01899 1 W10

K00814 1 W10

K03671 1 W10

K09458 1 W10

K02725 1 W10

E2.1.1 1 W10

K03236 1 W10

K04488 1 W10

K05993 1 W10

E3.6.5.3 1 W10

K00869 1 W10

E3.4.24.- 1 W10

K05610 1 W10

K03797 1 W10

K01465 1 W10

E1.14.11 1 W10

E3.1 1 W10

E3.6.4.1 1 W10

E3.6.1.3 1 W10

K06972 1 W10

K04523 1 W10

K02109 1 W11.61

K02109 1 W11.61

K02112 1 W11.61

K02112 1 W11.61

K02868 1 W11.61

K02868 1 W11.61

K02989 1 W11.61

E4.3.2.2 1 W11.61

K01441 1 W11.61

E2.3.1.48 1 W11.61

K00099 1 W11.61

K00022 1 W11.61

E2.4.1.34 1 W11.61

K01874 1 W11.61

K09499 1 W11.61

K01886 1 W11.61

E2.1.1.127 1 W11.61

K03531 1 W11.61

K07877 1 W11.61

K01840 1 W11.61

K02155 1 W11.61

E3.2.1.18 1 W11.61

E2.7.11.22 1 W11.61

K01148 1 W11.61

K01889 1 W11.61

K01072 1 W11.61

K02736 1 W11.61

E3.1.13.4 1 W11.61

E3.2.1.18 1 W11.61

K02868 1 W11.61

K00025 1 W11.61

K03943 1 W11.61

K03428 1 W11.61

K04802 1 W11.61

K03797 1 W11.61

K03061 1 W11.61

E3.6.5.3 1 W11.61

K01657 1 W11.61

K00260 1 W11.61

K00014 1 W11.61

K02964 1 W11.61

K00813 1 W11.61

K00955 1 W11.61

K02910 1 W11.61

K02973 1 W11.61

K03527 1 W11.61

E1 1 W11.61

K02917 1 W11.61

K01952 1 W11.61

K05808 1 W11.61

K00940 1 W11.61

K08902 1 W11.61

K02896 1 W11.61

K01938 1 W11.61

K07304 1 W11.61

K03070 1 W11.61

E3.1.2.16 1 W11.61

K08910 1 W11.61

K00942 1 W11.61

E1 1 W11.61

K00248 1 W11.61

K03531 1 W11.61

K00605 1 W11.61

K01358 1 W11.61

K09493 1 W11.61

K05915 1 W11.61

K02151 1 W11.61

K01625 1 W11.61

K01885 1 W11.61

E3.6.5.3 1 W11.61

K01951 1 W11.61

E1.2.4.1 1 W11.61

K01887 1 W11.61

K02975 1 W11.61

K00927 1 W11.61

E5.3.4.1 1 W11.61

K06942 1 W11.61

K01662 1 W11.61

K02891 1 W11.61

K00940 1 W11.61

K01529 1 W11.61

K06911 1 W11.61

K01599 1 W11.61

K03242 1 W11.61

K00945 1 W11.61

K02728 1 W11.61

K00232 1 W11.61

K00525 1 W11.61

K04508 1 W11.61

E1.14.11 1 W11.61

K02981 1 W12.92

K02981 1 W12.92

K02707 1 W12.92

K02707 1 W12.92

K02699 1 W12.92

K02699 1 W12.92

K02886 1 W12.92

K02985 1 W12.92

K00646 1 W12.92

E2.7.7 1 W12.92

K01676 1 W12.92

K02873 1 W12.92

K01175 1 W12.92

E1.9.3.1 1 W12.92

K01679 1 W12.92

K02912 1 W12.92

E2.1.1.45 1 W12.92

K01599 1 W12.92

K07976 1 W12.92

K03254 1 W12.92

K00366 1 W12.92

K02040 1 W12.92

K00088 1 W12.92

K00231 1 W12.92

K03686 1 W12.92

E3.4.24 1 W12.92

K04799 1 W12.92

K03064 1 W12.92

K06180 1 W12.92

K00799 1 W12.92

K03676 1 W12.92

K08776 1 W12.92

K00600 1 W12.92

K00295 1 W12.92

K02864 1 W12.92

K03036 1 W12.92

K09500 1 W12.92

K02889 1 W12.92

K01276 1 W12.92

K00926 1 W12.92

K00228 1 W12.92

E2.7.1.40 1 W12.92

K01890 1 W12.92

K02087 1 W12.92

K01529 1 W12.92

K00428 1 W12.92

K09503 1 W12.92

K00514 1 W12.92

E5.3.4.1 1 W12.92

K03062 1 W12.92

K07977 1 W12.92

K06942 1 W12.92

K02883 1 W12.92

K01939 1 W12.92

E1 1 W12.92

K01610 1 W12.92

K00162 1 W12.92

K06944 1 W12.92

K02693 1 W14.04

K03046 1 W14.04

K03046 1 W14.04

K02925 1 W14.04

K02993 1 W14.04

K01441 1 W14.04

K01992 1 W14.04

E3.2.1.18 1 W14.04

E2.5.1.47 1 W14.04

E1.2.4.1 1 W14.04

K00873 1 W14.04

E1 1 W14.04

K02949 1 W14.04

E3.6.3.14 1 W14.04

E3.1.3.16 1 W14.04

E6.3.2.- 1 W14.04

E1.11.1.7 1 W14.04

K08908 1 W14.04

K01915 1 W14.04

K00948 1 W14.04

K01256 1 W14.04

K02898 1 W14.04

E1.11.1.15 1 W14.04

K02731 1 W14.04

K00627 1 W14.04

E3.1.4.43 1 W14.04

K00161 1 W14.04

K03178 1 W14.04

K02974 1 W14.04

K00266 1 W14.04

K06118 1 W14.04

E3.6.1.3 1 W14.04

K01733 1 W14.04

K02991 1 W14.04

K00088 1 W14.04

E1.3.1.26 1 W14.04

E3.6.5.3 1 W14.04

K00003 1 W14.04

K02519 1 W14.04

K02726 1 W14.04

E3.6.1.3 1 W14.04

K03767 1 W14.04

K07976 1 W14.04

K03687 1 W14.04

K06212 1 W14.04

K01835 1 W14.04

K01803 1 W14.04

K00972 1 W14.04

E1.2.1.3 1 W14.04

K09488 1 W14.04

K02877 1 W14.04

K02870 1 W14.04

K07936 1 W14.04

K01710 1 W14.04

K07151 1 W14.04

E3 1 W14.04

K01855 1 W14.04

E2.1.1 1 W14.04

E3.4.21 1 W14.04

K01749 1 W14.04

K00939 1 W14.04

E2.1.1 1 W14.04

K02932 1 W15

E3.6.1.3 1 W15

K00930 1 W15

K01529 1 W15

K00145 1 W15

K01509 1 W15

E1 1 W15

E1 1 W15

E2.7.11.1 1 W15

K07976 1 W15

E2.3.1.48 1 W15

K03032 1 W15

K02894 1 W15

K03063 1 W15

K04499 1 W15

K01750 1 W15

K00698 1 W15

K02957 1 W15

K01256 1 W15

E1.1.1.18 1 W15

K02727 1 W15

K06445 1 W15

K09540 1 W15

K01254 1 W15

K00620 1 W15

K01755 1 W15

K02989 1 W15

K05864 1 W15

K03066 1 W15

K01358 1 W15

K06207 1 W15

K01778 1 W15

E1.2.1 1 W15

K00764 1 W15

K02882 1 W15

K02977 1 W15

K08738 1 W15

K01698 1 W15

K00627 1 W15

K07755 1 W15

E1.1.1.100 1 W15

K10413 1 W15

K02114 1 W15.85

K02114 1 W15.85

K02991 1 W15.85

E1.3.99.23 1 W15.85

K01961 1 W15.85

K02134 1 W15.85

E2.1.1 1 W15.85

K01868 1 W15.85

K07976 1 W15.85

K00908 1 W15.85

E1.8.1.4 1 W15.85

K02940 1 W15.85

K02969 1 W15.85

K01201 1 W15.85

K01933 1 W15.85

E1.1.1.31 1 W15.85

K02732 1 W15.85

K02150 1 W15.85

K01802 1 W15.85

E1 1 W15.85

K03942 1 W15.85

K02955 1 W15.85

K00101 1 W15.85

K01895 1 W15.85

K02149 1 W15.85

K01880 1 W15.85

K02900 1 W15.85

K01689 1 W15.85

K01803 1 W15.85

K02893 1 W15.85

K02690 1 W16.61

K02690 1 W16.61

K02870 1 W16.61

K02870 1 W16.61

K02635 1 W16.61

K02635 1 W16.61

K02930 1 W16.61

E3.2.1.3 1 W16.61

K01537 1 W16.61

K01624 1 W16.61

E2.3.1.48 1 W16.61

E2.7.1.150 1 W16.61

E1.4.1.4 1 W16.61

K02953 1 W16.61

K00297 1 W16.61

K02985 1 W16.61

K02729 1 W16.61

K00059 1 W16.61

K03405 1 W16.61

K00033 1 W16.61

K02641 1 W16.61

K01858 1 W16.61

K03253 1 W16.61

K02875 1 W16.61

K01869 1 W16.61

K04564 1 W16.61

E2.7.7 1 W16.61

K03065 1 W16.61

K02692 1 W17.3

K02692 1 W17.3

K03070 1 W17.3

K02479 1 W17.3

K02945 1 W17.3

K01586 1 W17.3

E3.6.-.- 1 W17.3

K01469 1 W17.3

E6.5.1.1 1 W17.3

E3.6.4.9 1 W17.3

E3.6.4.1 1 W17.3

K02981 1 W17.3

K00360 1 W17.3

K00963 1 W17.3

K00058 1 W17.3

K02987 1 W17.3

K01626 1 W17.3

K01687 1 W17.3

K00602 1 W17.3

K02866 1 W17.3

K02357 1 W17.3

K04078 1 W17.3

K01428 1 W17.3

K01069 1 W17.3

K01738 1 W17.3

K00382 1 W17.3

E3.4.24 1 W17.3

K01810 1 W17.3

K02993 1 W17.3

K03626 1 W17.3

K00820 1 W17.3

K01529 1 W17.3

K01711 1 W17.3

K01529 1 W17.3

K10046 1 W17.3

K00234 1 W17.3

E6.1.1.9 1 W17.3

K00873 1 W17.3

K02293 1 W17.3

K02115 1 W17.92

K02115 1 W17.92

K02634 1 W17.92

K02634 1 W17.92

K02703 1 W17.92

E3.4.24.64 1 W17.92

E3.2.1.3 1 W17.92

K02636 1 W17.92

K03028 1 W17.92

E2.5.1.47 1 W17.92

K01595 1 W17.92

K01953 1 W17.92

K01507 1 W17.92

K01873 1 W17.92

K00616 1 W17.92

K01834 1 W17.92

K04567 1 W17.92

K07374 1 W17.92

K10206 1 W17.92

K04564 1 W17.92

K02146 1 W17.92

K09565 1 W17.92

K02154 1 W17.92

K02872 1 W17.92

K02136 1 W17.92

K01897 1 W18.5

K01649 1 W18.5

K06959 1 W18.5

K06148 1 W18.5

K00600 1 W18.5

K02984 1 W18.5

E1 1 W18.5

K02937 1 W18.5

K08057 1 W18.5

K02960 1 W18.5

K06215 1 W18.5

K00855 1 W18.5

K00856 1 W18.5

K02932 1 W18.5

K02110 1 W19.04

K02110 1 W19.04

K00031 1 W19.04

K00430 1 W19.04

E2.5.1.22 1 W19.04

K00031 1 W19.04

K01955 1 W19.04

K00819 1 W19.04

K03252 1 W19.04

K02148 1 W19.04

K02908 1 W19.04

K01255 1 W19.04

K00208 1 W19.04

K03545 1 W19.04

K02962 1 W19.04

K00003 1 W19.04

K00325 1 W19.04

K00611 1 W19.04

K02720 1 W19.53

K01602 1 W19.53

K01602 1 W19.53

K00026 1 W19.53

K08769 1 W19.53

K02865 1 W19.53

K01835 1 W19.53

K02966 1 W19.53

E1.17.4.3 1 W19.53

K02901 1 W19.53

K02880 1 W19.53

K01870 1 W19.53

K00283 1 W19.53

K02705 1 W20

K02705 1 W20

K00958 1 W20

K03695 1 W20

K01696 1 W20

K01414 1 W20

K06028 1 W20

K02358 1 W20

K00818 1 W20

K02995 1 W20

K01903 1 W20

K03798 1 W20

K03969 1 W20

K01507 1 W20

K00052 1 W20

K02998 1 W20.44

K00058 1 W20.44

K02941 1 W20.44

K01624 1 W20.44

K09486 1 W20.44

K02031 1 W20.85

K00850 1 W20.85

E3.6.3.14 1 W20.85

E3.1.1.47 1 W20.85

K00134 1 W20.85

K02934 1 W20.85

E1.11.1.11 1 W20.85

K00927 1 W20.85

K00873 1 W20.85

K00012 1 W20.85

K02641 1 W20.85

K02706 1 W21.24

K02706 1 W21.24

K00820 1 W21.24

K01647 1 W21.24

K06972 1 W21.24

K01914 1 W21.24

K09291 1 W21.24

K00134 1 W21.24

K02137 1 W21.24

K07375 1 W21.24

E1 1 W21.61

K03695 1 W21.61

K00362 1 W21.61

K00134 1 W21.61

K02716 1 W21.61

K05236 1 W21.61

K01872 1 W21.61

K01940 1 W21.61

K02689 1 W21.96

K03798 1 W21.96

K04077 1 W21.96

K05862 1 W21.96

K00284 1 W21.96

K02936 1 W21.96

K01595 1 W21.96

K02355 1 W22.3

K05692 1 W22.3

K08912 1 W22.3

K07977 1 W22.3

K01689 1 W22.3

K04077 1 W22.62

K01897 1 W22.62

K02641 1 W22.62

K00615 1 W22.62

K06413 1 W22.62

K03696 1 W22.92

K03696 1 W22.92

K03696 1 W22.92

E1.11.1.11 1 W22.92

K01959 1 W22.92

K09485 1 W22.92

K02115 1 W22.92

K01845 1 W22.92

K03257 1 W22.92

K02930 1 W23.22

K01829 1 W23.22

K02925 1 W23.22

K03405 1 W23.5

K03405 1 W23.5

K03403 1 W23.5

K00265 1 W23.5

K01703 1 W23.5

E3.6.1.3 1 W23.77

K02147 1 W23.77

K00548 1 W23.77

K06630 1 W24.29

K05863 1 W24.29

K04043 1 W24.53

K00053 1 W24.77

K00789 1 W25

K01682 1 W25

K01552 1 W25

K04077 1 W25

K00392 1 W25.22

K05907 1 W25.22

K02145 1 W25.22

K04079 1 W25.22

K00956 1 W25.65

K01624 1 W25.85

K01955 1 W26.05

K02704 1 W26.43

K02704 1 W26.43

K02132 1 W26.61

K03235 1 W27.13

K04043 1 W27.13

K00134 1 W27.46

K03234 1 W27.77

K09490 1 W27.92

E3.2.1.3 1 W28.36

K00615 1 W28.64

K01251 1 W28.64

K01915 1 W29.16

K02358 1 W29.29

E3.6.3.14 1 W29.65

E3.6.3.14 1 W29.65

K02133 1 W29.65

K00927 1 W29.77

K04646 1 W30.11

K01601 1 W30.44

K01601 1 W30.44

K02112 1 W30.95

K03283 1 W32.05

K01961 1 W32.05

*

*

*

*

*

*

Fe replete data- copy list below (end at the *) do not change formatting

K02112 1 W31.96

E3.6.3.14 1 W31.05

E3.6.3.14 1 W31.05

K03283 1 W30.85

K01915 1 W30.54

K01601 1 W30.11

K01601 1 W30.11

K01961 1 W30.11

K02133 1 W29.16

K02704 1 W28.64

K02704 1 W28.64

K03235 1 W28.64

K04646 1 W27.77

E3.2.1.3 1 W27.62

K02132 1 W27.62

K02358 1 W27.3

K00615 1 W27.13

K09490 1 W26.61

K01251 1 W26.24

K04043 1 W26.05

K00927 1 W26.05

K01552 1 W25.85

K01955 1 W25.85

K02689 1 W25

K04077 1 W25

K03798 1 W24.53

K00956 1 W24.53

K03234 1 W24.29

K02115 1 W24.29

K05862 1 W24.04

K00134 1 W24.04

K05863 1 W24.04

K02930 1 W23.5

K01682 1 W23.5

K02995 1 W23.5

K02716 1 W23.5

E1 1 W23.22

K05907 1 W23.22

K04079 1 W23.22

K02870 1 W22.92

K02870 1 W22.92

K03696 1 W22.92

K03696 1 W22.92

K03696 1 W22.92

K00053 1 W22.92

K02641 1 W22.92

K03403 1 W22.92

K02934 1 W22.92

K02147 1 W22.92

K02706 1 W22.62

K02706 1 W22.62

K04043 1 W22.62

K02925 1 W22.62

K02962 1 W22.62

K02705 1 W22.3

K02705 1 W22.3

K00392 1 W22.3

K01959 1 W22.3

K02115 1 W21.96

K02115 1 W21.96

K02634 1 W21.96

K02634 1 W21.96

K01647 1 W21.96

K00362 1 W21.96

K02145 1 W21.96

K01829 1 W21.61

E3.6.3.14 1 W21.24

K01897 1 W21.24

K02941 1 W21.24

K00265 1 W21.24

K01624 1 W21.24

K02720 1 W20.85

K02703 1 W20.85

E3.6.1.3 1 W20.85

K02936 1 W20.85

K00134 1 W20.85

K02137 1 W20.85

K03798 1 W20.85

K01689 1 W20.85

K02641 1 W20.85

K02690 1 W20.44

K02690 1 W20.44

K04077 1 W20.44

K02981 1 W20.44

K02355 1 W20.44

E3.1.1.47 1 W20.44

K00134 1 W20.44

K02901 1 W20.44

K01870 1 W20.44

K01703 1 W20.44

K01845 1 W20.44

K00548 1 W20.44

K00789 1 W20

K02998 1 W20

K02031 1 W20

K00850 1 W20

K04077 1 W20

K02636 1 W20

K01595 1 W20

K02358 1 W20

K01595 1 W20

K02993 1 W20

K01529 1 W20

K00820 1 W19.53

K02945 1 W19.53

K08912 1 W19.53

K03252 1 W19.53

K03405 1 W19.04

K03405 1 W19.04

K02932 1 W19.04

E1.3.99.23 1 W19.04

E3.2.1.3 1 W19.04

K00360 1 W19.04

K05692 1 W19.04

K00284 1 W19.04

K02987 1 W19.04

K02966 1 W19.04

K02880 1 W19.04

K00101 1 W19.04

K01507 1 W19.04

K00325 1 W19.04

K02110 1 W18.5

K02110 1 W18.5

K02930 1 W18.5

K02886 1 W18.5

K01529 1 W18.5

K02865 1 W18.5

K00058 1 W18.5

K02937 1 W18.5

K00602 1 W18.5

K00283 1 W18.5

K02136 1 W18.5

K02932 1 W18.5

K03257 1 W18.5

K01602 1 W17.92

K01602 1 W17.92

K02692 1 W17.92

K02692 1 W17.92

K02989 1 W17.92

K02134 1 W17.92

K08769 1 W17.92

K00430 1 W17.92

E3.6.-.- 1 W17.92

K01914 1 W17.92

K02984 1 W17.92

K02969 1 W17.92

E1.11.1.11 1 W17.92

K06180 1 W17.92

K02866 1 W17.92

E1 1 W17.92

K02875 1 W17.92

K00820 1 W17.92

K02154 1 W17.92

K02872 1 W17.92

K03046 1 W17.3

K03046 1 W17.3

K02991 1 W17.3

K01897 1 W17.3

K00026 1 W17.3

E3.6.4.9 1 W17.3

K03255 1 W17.3

K01955 1 W17.3

K02908 1 W17.3

K01903 1 W17.3

K09485 1 W17.3

K06413 1 W17.3

K02882 1 W17.3

K01872 1 W17.3

K01939 1 W17.3

K06215 1 W17.3

K02109 1 W16.61

K02109 1 W16.61

K02981 1 W16.61

K02981 1 W16.61

K02925 1 W16.61

K02993 1 W16.61

K00031 1 W16.61

K01537 1 W16.61

K06959 1 W16.61

E6.5.1.1 1 W16.61

K06148 1 W16.61

K02949 1 W16.61

K06972 1 W16.61

E3.4.24.64 1 W16.61

K02894 1 W16.61

K06630 1 W16.61

K03695 1 W16.61

K06028 1 W16.61

K02964 1 W16.61

K02974 1 W16.61

K02991 1 W16.61

K01834 1 W16.61

K00819 1 W16.61

K02985 1 W16.61

K04078 1 W16.61

K00831 1 W16.61

K03253 1 W16.61

K00514 1 W16.61

K02146 1 W16.61

K07977 1 W16.61

K02960 1 W16.61

K01940 1 W16.61

K00611 1 W16.61

K02693 1 W15.85

K02868 1 W15.85

K02868 1 W15.85

K03043 1 W15.85

K03043 1 W15.85

K02635 1 W15.85

K02635 1 W15.85

K02479 1 W15.85

K00031 1 W15.85

K01750 1 W15.85

K02940 1 W15.85

K03254 1 W15.85

K02953 1 W15.85

K02040 1 W15.85

K02885 1 W15.85

K01733 1 W15.85

K02519 1 W15.85

K02150 1 W15.85

E1.17.4.3 1 W15.85

K02989 1 W15.85

K03405 1 W15.85

K01624 1 W15.85

K00927 1 W15.85

K00003 1 W15.85

K09486 1 W15.85

K00234 1 W15.85

K00052 1 W15.85

K02891 1 W15.85

K02985 1 W15

K01441 1 W15

K01961 1 W15

K02956 1 W15

E2.7.1.150 1 W15

K09291 1 W15

K03797 1 W15

K00266 1 W15

K09540 1 W15

K00955 1 W15

K00600 1 W15

K02910 1 W15

K02357 1 W15

K01516 1 W15

K06212 1 W15

K08057 1 W15

E1.11.1.5 1 W15

K00382 1 W15

K09503 1 W15

E5.3.4.1 1 W15

K02900 1 W15

K00873 1 W15

K02883 1 W15

E3.6.1.- 1 W15

K02893 1 W15

K00856 1 W15

E1.1.1.100 1 W15

K02964 1 W14.04

K02964 1 W14.04

K02114 1 W14.04

K02114 1 W14.04

K03070 1 W14.04

E3.6.1.3 1 W14.04

K00958 1 W14.04

K01649 1 W14.04

E1 1 W14.04

K02873 1 W14.04

E3.2.1.3 1 W14.04

E3.6.4.1 1 W14.04

K00908 1 W14.04

E1.8.1.4 1 W14.04

K02912 1 W14.04

K03428 1 W14.04

K01835 1 W14.04

K00058 1 W14.04

K06118 1 W14.04

K00818 1 W14.04

K01687 1 W14.04

K02973 1 W14.04

E1.3.1.26 1 W14.04

K01255 1 W14.04

K01428 1 W14.04

E3.6.1.3 1 W14.04

K02955 1 W14.04

K02876 1 W14.04

K07375 1 W14.04

K08902 1 W14.04

K06207 1 W14.04

E1.2.1 1 W14.04

K02877 1 W14.04

K06185 1 W14.04

K08738 1 W14.04

K00627 1 W14.04

K01529 1 W14.04

K02112 1 W12.92

K02112 1 W12.92

K03046 1 W12.92

K02707 1 W12.92

K02707 1 W12.92

K02694 1 W12.92

K02694 1 W12.92

K02949 1 W12.92

K03237 1 W12.92

E3.2.1.18 1 W12.92

K10251 1 W12.92

E2.3.1.48 1 W12.92

E1.9.3.1 1 W12.92

K01696 1 W12.92

K02868 1 W12.92

K09838 1 W12.92

E1.10.2.2 1 W12.92

K00963 1 W12.92

K03028 1 W12.92

K02957 1 W12.92

K02938 1 W12.92

K01414 1 W12.92

K00615 1 W12.92

K00627 1 W12.92

K00161 1 W12.92

E1 1 W12.92

K00295 1 W12.92

K05679 1 W12.92

K00134 1 W12.92

E3.6.5.3 1 W12.92

K02889 1 W12.92

K01802 1 W12.92

K03942 1 W12.92

K02729 1 W12.92

K05808 1 W12.92

K10206 1 W12.92

K01738 1 W12.92

K04564 1 W12.92

K00344 1 W12.92

K03545 1 W12.92

E2.1.1.95 1 W12.92

E3.4.24 1 W12.92

E1.11.1.11 1 W12.92

K02870 1 W12.92

K01529 1 W12.92

K01711 1 W12.92

K02977 1 W12.92

K07151 1 W12.92

E6.1.1.9 1 W12.92

E5.3.4.1 1 W12.92

E2.1.1 1 W12.92

E3.4.21 1 W12.92

K01749 1 W12.92

E1.14.19.3 1 W12.92

K05665 1 W12.92

K02125 1 W11.61

K03936 1 W11.61

E1.5.1.2 1 W11.61

E1.1.1.18 1 W11.61

E2.7.7 1 W11.61

E2.1.1 1 W11.61

E2.5.1.22 1 W11.61

E2.7.11.1 1 W11.61

K02155 1 W11.61

E2.7.11.22 1 W11.61

K07976 1 W11.61

K03076 1 W11.61

E1.3.99.23 1 W11.61

K08908 1 W11.61

K00948 1 W11.61

E1.4.1.4 1 W11.61

K02898 1 W11.61

K09272 1 W11.61

K01201 1 W11.61

E3.6.5.3 1 W11.61

K03686 1 W11.61

E3.1.4.43 1 W11.61

E1.3.1.- 1 W11.61

K01507 1 W11.61

K02864 1 W11.61

K01626 1 W11.61

E2.7.11.22 1 W11.61

K00872 1 W11.61

E1 1 W11.61

K02917 1 W11.61

K02983 1 W11.61

K00939 1 W11.61

K03066 1 W11.61

K00235 1 W11.61

K07976 1 W11.61

K00324 1 W11.61

K08910 1 W11.61

K05236 1 W11.61

K08906 1 W11.61

K03062 1 W11.61

K03626 1 W11.61

K04564 1 W11.61

K01803 1 W11.61

K00012 1 W11.61

K02975 1 W11.61

K00873 1 W11.61

K03065 1 W11.61

K01610 1 W11.61

K01698 1 W11.61

K01662 1 W11.61

K02293 1 W11.61

E3.6.5.3 1 W11.61

K01599 1 W11.61

K03242 1 W11.61

K00162 1 W11.61

E3.1 1 W11.61

E3.2.1.18 1 W11.61

K02709 1 W10

K02709 1 W10

K02699 1 W10

K02699 1 W10

K02997 1 W10

K00930 1 W10

K02903 1 W10

E3.2.1.18 1 W10

K01586 1 W10

K01624 1 W10

E3.2.1.3 1 W10

E6.3.2.19 1 W10

K03695 1 W10

K01679 1 W10

K02736 1 W10

K04499 1 W10

E3.1.-.- 1 W10

K01836 1 W10

K01599 1 W10

K03100 1 W10

K02731 1 W10

K02732 1 W10

K02727 1 W10

K01953 1 W10

E3.6.1.3 1 W10

K00164 1 W10

K00297 1 W10

K00088 1 W10

K01834 1 W10

K02148 1 W10

K01276 1 W10

K07374 1 W10

K01952 1 W10

K02726 1 W10

K00940 1 W10

E2.7.1.40 1 W10

K03969 1 W10

K02896 1 W10

K03687 1 W10

K00326 1 W10

K02641 1 W10

K01637 1 W10

K01529 1 W10

K01358 1 W10

K03238 1 W10

K01803 1 W10

K02879 1 W10

K03455 1 W10

K02149 1 W10

K07936 1 W10

K01689 1 W10

K03038 1 W10

K08956 1 W10

K10046 1 W10

K02918 1 W10

K00855 1 W10

K05805 1 W10

K01358 1 W10

K01758 1 W10

K04488 1 W10

K00945 1 W10

K02013 1 W10

E3.1.3.2 1 W10

E1.14.11 1 W10

K02723 1 W7.92

K02723 1 W7.92

K02891 1 W7.92

K02891 1 W7.92

K02256 1 W7.92

K02261 1 W7.92

E2.7.1.- 1 W7.92

K01441 1 W7.92

E2.3.1.48 1 W7.92

K02730 1 W7.92

E2.4.1.34 1 W7.92

E5.3.4.1 1 W7.92

K03531 1 W7.92

K01829 1 W7.92

K02947 1 W7.92

E2.3.1.12 1 W7.92

K00329 1 W7.92

E1 1 W7.92

K02971 1 W7.92

E1 1 W7.92

E2 1 W7.92

K01868 1 W7.92

K07976 1 W7.92

K01175 1 W7.92

K03937 1 W7.92

E3.6.5.3 1 W7.92

E2.7.11.22 1 W7.92

K09496 1 W7.92

K03940 1 W7.92

E3.1.3.16 1 W7.92

K00820 1 W7.92

E1.11.1.7 1 W7.92

E3.1.13.4 1 W7.92

E3.4.21.4 1 W7.92

E1.3.1.33 1 W7.92

K01915 1 W7.92

K03031 1 W7.92

K07976 1 W7.92

K04040 1 W7.92

K00226 1 W7.92

K00698 1 W7.92

E1.14.-.- 1 W7.92

E1.11.1.15 1 W7.92

E1.1.1.31 1 W7.92

K07517 1 W7.92

K03696 1 W7.92

K02997 1 W7.92

K09838 1 W7.92

K00813 1 W7.92

K03064 1 W7.92

K01254 1 W7.92

K00799 1 W7.92

K01873 1 W7.92

K00698 1 W7.92

K08869 1 W7.92

K01755 1 W7.92

K03264 1 W7.92

E1 1 W7.92

K00059 1 W7.92

K03767 1 W7.92

K02958 1 W7.92

K03100 1 W7.92

K00942 1 W7.92

K01802 1 W7.92

K00134 1 W7.92

K00559 1 W7.92

K01858 1 W7.92

K01358 1 W7.92

K09493 1 W7.92

K09560 1 W7.92

K02951 1 W7.92

K02151 1 W7.92

K01810 1 W7.92

K01625 1 W7.92

K02979 1 W7.92

K01880 1 W7.92

K07977 1 W7.92

E3.6.5.3 1 W7.92

K01090 1 W7.92

K03263 1 W7.92

K01951 1 W7.92

K05665 1 W7.92

K01887 1 W7.92

K01885 1 W7.92

E2.7.7 1 W7.92

E3.6.1.- 1 W7.92

E3.6.1.3 1 W7.92

K06942 1 W7.92

K00592 1 W7.92

K03754 1 W7.92

K07755 1 W7.92

K02734 1 W7.92

K01054 1 W7.92

K01325 1 W7.92

K00939 1 W7.92

E2.1.1 1 W7.92

K03241 1 W7.92

K02921 1 W7.92

K02728 1 W7.92

E3.4.24.- 1 W7.92

E5.2.1.8 1 W7.92

K00232 1 W7.92

K02357 1 W7.92

K03627 1 W7.92

K06158 1 W7.92

E2.1.1.127 1 W7.92

E2.3.1.74 1 W7.92

K10413 1 W7.92

K02999 1 W7.92

K00111 1 W7.92

K02469 1 W7.92

K02541 1 W7.92

K02040 1 W5

K03935 1 W5

K02837 1 W5

K00022 1 W5

K00940 1 W5

E2.5.1.47 1 W5

K01262 1 W5

K09499 1 W5

E4.1.1.29 1 W5

E2.1.1.127 1 W5

K04774 1 W5

K06889 1 W5

E3.6.3.16 1 W5

K03839 1 W5

K02976 1 W5

K02943 1 W5

E6.3.2.- 1 W5

K01469 1 W5

K01423 1 W5

E1 1 W5

K01840 1 W5

K00873 1 W5

E1 1 W5

E3.2.1.18 1 W5

E2.3.1.48 1 W5

E3.6.3.14 1 W5

K01165 1 W5

K03032 1 W5

E6.3.2.- 1 W5

K03063 1 W5

K06980 1 W5

E2.3.1.48 1 W5

K00927 1 W5

K09498 1 W5

E3.2.1.18 1 W5

E3.6.5.3 1 W5

E5.4.2.8 1 W5

K01931 1 W5

K03943 1 W5

K04518 1 W5

K04802 1 W5

K01897 1 W5

E1.11.1.7 1 W5

E2.7.1.137 1 W5

E1.1.1.- 1 W5

K00387 1 W5

K03061 1 W5

K03953 1 W5

K01256 1 W5

K01086 1 W5

E3.6.3.14 1 W5

K01933 1 W5

K03246 1 W5

E1.3.1.33 1 W5

K06063 1 W5

K06445 1 W5

K03531 1 W5

E3.6.5.3 1 W5

K03676 1 W5

E3.6.1.3 1 W5

K00620 1 W5

E1.14.11 1 W5

K03076 1 W5

K00698 1 W5

K00003 1 W5

K02978 1 W5

K01069 1 W5

E1.10.2.2 1 W5

E1.10.2.2 1 W5

K03977 1 W5

K00133 1 W5

E3.6.1.3 1 W5

K01890 1 W5

K07304 1 W5

K00033 1 W5

K03955 1 W5

K03030 1 W5

K01802 1 W5

K03037 1 W5

E3.4.11.18 1 W5

K01895 1 W5

E3.1.2.16 1 W5

E5.2.1.8 1 W5

K01652 1 W5

K03257 1 W5

K01476 1 W5

K00632 1 W5

K03627 1 W5

K03033 1 W5

K07942 1 W5

E1.2.1.3 1 W5

K03249 1 W5

K00301 1 W5

E1 1 W5

K01885 1 W5

K09488 1 W5

K03262 1 W5

K00789 1 W5

K06067 1 W5

K01710 1 W5

K01899 1 W5

K00814 1 W5

K03671 1 W5

E3.2.1.3 1 W5

K00927 1 W5

K00413 1 W5

E1.3.1.33 1 W5

K01855 1 W5

E3.6.5.3 1 W5

K01362 1 W5

K08023 1 W5

K07976 1 W5

E3.2.1.18 1 W5

K02959 1 W5

K02924 1 W5

K03177 1 W5

K03439 1 W5

E1.14.11 1 W5

K01187 1 W5

K03097 1 W5

E5.2.1.8 1 W5

E3.6.1.3 1 W5

K08869 1 W5

K00624 1 W5

K10356 1 W5

K06119 1 W5

K06948 1 W5

K01803 1 W5

K06067 1 W5

K03798 1 W5

K01872 1 W5

E5.2.1.8 1 W5

E6.3.2.- 1 W5

E2.7.1.37 1 W5

E4 1 W5

K03879 1 W5

K01611 1 W5

E3.1.13.- 1 W5

K01875 1 W5

K06924 1 W5

E5.2.1.8 1 W5

K10226 1 W5

K08869 1 W5

K03797 1 W5

K10256 1 W5

E2.7.7.6 1 W5
